# E2F1-Driven WDHD1 Transcription Enhances Cell-Cycle Progression and Promotes Pancreatic Cancer Progression

**DOI:** 10.3390/curroncol33040186

**Published:** 2026-03-26

**Authors:** Xiaojuan Yang, Zhiwei Zhang, Shuangjuan Lv, Buzhe Zhang, Xue Tao, Chang Liu, Qing Zhu

**Affiliations:** 1 Department of Abdominal Oncology, West China Hospital, Sichuan University, Chengdu 610041, China; y3532388972@163.com (X.Y.); 12418716@zju.edu.cn (Z.Z.); 2 Laboratory of Hepatic AI Translation, Frontiers Science Center for Disease-Related Molecular Network, West China Hospital, Sichuan University, Chengdu 610041, China; lvshuangjuan@wchscu.cn (S.L.); 2024224020203@stu.scu.edu.cn (B.Z.); 2024224200031@stu.scu.edu.cn (X.T.); 3 Key Laboratory of Cancer Prevention and Intervention, Department of Colorectal Surgery and Oncology, Ministry of Education, the Second Affiliated Hospital, Zhejiang University School of Medicine, Hangzhou 322000, China; 4 Intervention Center, West China Hospital, Sichuan University, No. 37 Guoxue Alley, Chengdu 610041, China

**Keywords:** pancreatic cancer, E2F1, WDHD1, cell cycle, tumor proliferation

## Abstract

*WD repeat and HMG-box DNA-binding protein 1 (WDHD1)* has been identified as a crucial oncogene in various tumors, but its role in pancreatic cancer remains unexplored. This study investigated the mechanisms by which WDHD1 contributes to pancreatic cancer progression. Differential analysis of the Cancer Genome Atlas (TCGA) pancreatic cancer samples identified abnormally expressed genes. Cellular assays, including cell proliferation, flow cytometry, and apoptosis assays, demonstrated WDHD1’s oncogenic role. WDHD1 expression was significantly elevated in pancreatic cancer cells and tissues compared to normal counterparts. Knockdown of WDHD1 inhibited cell proliferation, induced apoptosis, and caused G1-phase cell-cycle arrest. *In vivo* xenograft models further validated that WDHD1 knockdown suppressed the growth of pancreatic cancer cells. Mechanistically, WDHD1 knockdown resulted in significant reductions in CDK4 and cyclin D1 protein levels, whereas WDHD1 overexpression produced the opposite effects. Additionally, E2F1 overexpression increased the expression of *WDHD1* at both mRNA and protein levels. Rescue experiments revealed that WDHD1 knockdown could reverse the E2F1-induced upregulation of CDK4 and cyclin D1 protein levels. In conclusion, E2F1 promotes pancreatic cancer cell proliferation and cell-cycle progression by upregulating WDHD1, which in turn enhances the expression of the CDK4–cyclin D1 complex.

## Simple Summary

*WD repeat and HMG-box DNA-binding protein 1 (WDHD1)* has been identified as a key oncogene in several tumors, but its role in pancreatic cancer is unclear. We examined WDHD1’s function and mechanisms in pancreatic cancer. Analysis of the Cancer Genome Atlas showed WDHD1 upregulation, which we confirmed in tumor tissues and cell lines. Functionally, WDHD1 knockdown inhibited proliferation, induced apoptosis, caused G1-phase arrest *in vitro*, and suppressed tumor growth in xenograft models. Mechanistically, WDHD1 depletion reduced CDK4 and cyclin D1 protein levels, whereas overexpression had the opposite effect. E2F1 is a direct transcriptional activator of WDHD1: E2F1 overexpression increased *WDHD1* mRNA and protein, and WDHD1 knockdown abrogated E2F1-driven upregulation of CDK4 and cyclin D1. Thus, E2F1 promotes pancreatic cancer cell proliferation and cell-cycle progression by upregulating WDHD1, which enhances the CDK4–cyclin D1 complex, highlighting WDHD1 as a potential therapeutic target.

## 1. Introduction

Tumor initiation and progression remain central, yet challenging frontiers in cancer research. Elucidating the key proteins that drive these processes will accelerate drug discovery and spur therapeutic innovation. Pancreatic cancer, a highly refractory malignancy, is marked by a grim 5-year survival rate below 10%, with curatively resected patients seldom exceeding a 20% long-term survival threshold [1,2]. Projections indicate it will become the second-leading cause of cancer-related death worldwide by 2030 [3], owing to poor early detection, the predominance of late-stage presentation, and the limited eligibility for curative surgery [4]. Its intrinsic aggressiveness leads to frequent recurrence and poor outcomes even after treatment, with median survival after surgical resection of only 12–25 months [5]. These observations underscore the limitations of current interventions and the urgent need for new therapies. Defining the mechanisms that drive pancreatic cancer initiation and progression—and leveraging these insights to refine treatment—is therefore imperative.

WD repeat and HMG-box DNA-binding protein 1 (WDHD1) is a DNA-binding protein characterized by a tripartite structure: an N-terminus with WD40 repeats for protein-peptide interactions, a central SepB domain, and a C-terminal high-mobility group (HMG) box domain [6]. WD40 repeats mediate diverse cellular processes, including signaling pathway regulation, chromatin remodeling, and the DNA damage response, by providing extensive protein–protein interaction surfaces [7]. WDHD1, an integral component of the DNA replication machinery, maintains replication-fork stability and fidelity, thereby ensuring efficient and accurate genome duplication. It also functions in the DNA damage response, where it is recruited to DNA lesions and helps orchestrate cellular responses to replication stress [8]. Furthermore, by promoting Claspin accumulation at stalled replication forks, WDHD1 enables efficient CDK1 activation—a key step in preserving replication-fork integrity [9].

Studies indicate that WDHD1 is overexpressed and exhibits oncogenic functions in breast cancer [10], non-small-cell lung cancer [11], and esophageal squamous cell carcinoma [12]. However, its expression, function, and specific mechanisms of action in pancreatic cancer have not yet been explored. This study investigated WDHD1’s role and regulatory mechanisms in pancreatic cancer development and progression, offering experimental support for the potential clinical use of WDHD1 inhibitors.

## 2. Materials and Methods

### 2.1. Cell Culture

We obtained the pancreatic cancer cell lines PANC-1, CFPAC-1, and 293T from the Shanghai Institute of Cell Biology, Chinese Academy of Sciences. The hTERT-HPNE, MIA-PaCa-2, AsPC-1, and BxPC3 cell lines were obtained from the BNCC cell repository and authenticated by short tandem repeat (STR) profiling on 20 July 2021 prior to ethics approval and study initiation. The cell lines were cultured in Dulbecco’s modified Eagle medium (HyClone), RPMI-1640 medium (HyClone), or IMDM medium (Gibco) supplemented with 10% fetal bovine serum, 2 mmol/L glutamine, 100 U/mL penicillin, and 100 mg/mL streptomycin at 37 °C in a 5% CO_2_ atmosphere. All cell lines were reinitiated from the original stocks at intervals of no more than two months. Cultures were routinely screened for mycoplasma and examined by microscopy for morphological changes.

### 2.2. Western Blot Analysis

Total cellular protein was extracted in RIPA buffer. Equal amounts of protein were resolved by SDS–PAGE (10% or 12.5%) and transferred to PVDF membranes (Millipore). Membranes were blocked with 5% non-fat milk in TBST for 1 h at room temperature and incubated overnight at 4 °C with primary antibodies against WDHD1, E2F1, CDK4, CDK6, cyclin D1, cyclin D3 and β-actin (antibody sources, catalog numbers, and working dilutions are provided in Section 2.6). After washing in TBST, membranes were incubated with HRP-conjugated secondary antibodies (ZSGB-BIO, Beijing, China, ZB-2301 and ZB-2305) for 2 h at room temperature. Signals were detected by enhanced chemiluminescence (Millipore, Burlington, MA, USA). Immunoblots shown are representative of three independent experiments.

### 2.3. Quantitative Real-Time Polymerase Chain Reaction (qRT-PCR) and PCR

Total RNA was isolated from human pancreatic cancer tissues and cell lines using a Cell Total RNA isolation kit (Foregene, RE-03111, Chengdu, China). cDNA was synthesized with HiScript III RT SuperMix (Vazyme, Nanjing, China) following the manufacturer’s instructions. Quantitative real-time PCR (qPCR) was performed using ChamQ SYBR qPCR Master Mix (Vazyme). GAPDH was used as the reference gene, and relative mRNA abundance was calculated using the 2^−ΔΔCt^ method. Primer sequences are provided in Supplementary Table S1.

### 2.4. Cell Proliferation Detection

Cell proliferation was measured using the Cell Counting Kit-8 (CCK-8, Oriscience Biotechnology, Chengdu, China) assay [13,14,15]. Tumor cells were dissociated with 0.25% trypsin, resuspended at 4 × 10^4^ cells/mL, and seeded into 96-well plates at 100 µL per well (4 × 10^3^ cells per well), with six technical replicates per condition. At 24, 48, 72, and 96 h after seeding, 10 µL of CCK-8 reagent was added to each well and plates were incubated for 2 h at 37 °C in 5% CO_2_. Absorbance at 450 nm was recorded on an Epoch 2 microplate reader (BioTek, Winooski, VT, USA).

Clonogenic potential was assessed by colony formation assay. Log-phase cells were transfected as indicated, allowed to recover, and dissociated with 0.25% trypsin. Viable (trypan blue–negative) cells were counted and plated in six-well plates at 2000 cells per well in complete medium, with replicate wells per condition. Early attachment and viability were verified 6–8 h after seeding to ensure comparable plating across groups. Cultures were maintained for 2–3 weeks, with media changed every 2–3 days, until visible colonies formed. Colonies were fixed with 4% paraformaldehyde (PFA) (in PBS) for 30 min, stained with crystal violet for 30 min, rinsed three times with distilled water, air-dried, and colonies containing > 50 cells were counted. Plating efficiency was calculated as the number of colonies divided by the number of cells seeded, and surviving fractions were obtained by normalizing each plating efficiency to the corresponding control. This protocol follows our previously described methods [16].

### 2.5. Construction of shRNAs and Plasmids

shRNA oligos targeting *WDHD1* (Table 1) were cloned into the pLKO.1-TRC vector and co-transfected into HEK-293T cells with the packaging plasmids psPAX2 and pMD2.G to generate the *WDHD1* knockdown virus. Pancreatic cancer cells were infected with the virus for 48 h and subsequently selected in media containing 2.5 μg/mL puromycin until achieving 80–90% confluency of positively infected cells. Human *WDHD1* and *E2F1* genes were amplified by PCR and cloned into the plenti-CMV-3HA vector, or pcDNA3.1-3Flag. This recombinant plasmid was transfected into pancreatic cancer cell lines using polyethylenimine (PEI) or lentivirus. All plasmid constructs were verified by DNA sequencing, and their expression efficiency was confirmed by reverse-transcription PCR (RT-PCR) and/or Western blot analysis.

### 2.6. Antibodies

The antibodies utilized in this study included anti-WDHD1 (abclonal, A15396, 1:1000), anti-E2F1 (abclonal, A19579, 1:1000), anti-CDK4 (abclonal, A0366, 1:1000), anti-CDK6 (abclonal, A1545, 1:1000), anti-cyclin D1 (abclonal, A1301, 1:1000), and anti-cyclin D3 (abclonal, A3589, 1:1000). β-actin (ZSGB-BIO, TA-09, 1:1000) was used as the sample loading control.

### 2.7. EdU Incorporation Assay

Cell proliferation was assessed using a 5-ethynyl-2′-deoxyuridine (EdU) incorporation assay kit (RiboBio, Guangzhou, China, C10310-3). Briefly, treated cells were incubated with 50 µmol/L EdU at 37 °C for 3 h. Cells were then fixed with 4% PFA and permeabilized with 0.3% Triton X-100 solution. Following this, cells were stained with 1× Click reaction solution for 30 min and subsequently counterstained with Hoechst 33342 for 30 min. Hoechst and EdU-positive cells were observed using a Nikon STORM super-resolution microscope (Nikon A1, Tokyo, Japan).

### 2.8. Cell Cycle Analysis

Cell-cycle assays were performed using a cell-cycle staining kit (FXP0211, 4A BIOTECH, Beijing, China) following the manufacturer’s protocol [17]. To begin the process, five million cells were seeded into each well of a six-well plate. At 72 h post-transfection, the cells were harvested and subjected to two washes with PBS to remove any residual media or debris. Following the washes, the cells were fixed overnight in 70% ethanol at 4 °C, which helps to preserve cellular structures and permeabilize the cells for subsequent staining. After the overnight fixation, the cells were treated with RNAase at 37 °C for 30 min to eliminate RNA, which could otherwise interfere with the PI staining by binding to the dye. The cells were then stained with PI for an additional 30 min at 4 °C in the dark to prevent photobleaching and ensure optimal staining conditions. PI intercalates into the DNA, allowing for the quantification of DNA content within the cells, which is indicative of their position within the cell cycle. The stained cells were subsequently analyzed using BD Pharmingen flow cytometers (BD Pharmingen, Franklin Lakes, NJ, USA).

All cell-cycle analyses were performed in FlowJo v10.8.1. Briefly, intact cells were gated on FSC-A versus SSC-A to exclude debris, doublets/aggregates were removed using pulse-geometry gates (FSC-H vs. FSC-A and PI-W vs. PI-A) to retain singlets, and cell-cycle distributions were quantified from the singlet population using PI-A DNA-content histograms.

### 2.9. Apoptosis Assay

An annexin V–FITC/PI apoptosis kit from 4A BIOTECH, China was employed to accurately quantify the proportion of apoptotic cells. The procedure began with the trypsinization of adherent cells to detach them from the culture surface. Following trypsinization, the cells were carefully washed with PBS to remove any remaining enzymes and detached debris, ensuring clean cell preparation for subsequent steps. Next, the washed cells were resuspended in 500 μL of binding buffer, which is essential for maintaining the appropriate conditions for annexin V–FITC binding. The, 10 μL of FITC-conjugated annexin V and 5 μL of PI were added to the cell suspensions. Annexin V–FITC binds to phosphatidylserine residues that translocate to the outer leaflet of the plasma membrane early in apoptosis, while PI stains the DNA of cells with compromised membranes, typically late apoptotic and necrotic cells. Following the addition of the staining reagents, the cell suspensions were incubated for 5 min in the dark. This step is crucial to prevent photobleaching of the fluorescent dyes and to ensure accurate staining. After the incubation period, the stained cells were immediately analyzed using a BD Pharmingen flow cytometer (BD Pharmingen, Franklin Lakes, NJ, USA).

### 2.10. Immunofluorescence

Cells were seeded onto coverslips in a 24-well plate at a density of 2 × 10^4^ cells per well. After 24 h of culture, the cells were fixed with 4% PFA for 15 min, followed by permeabilization with 0.5% Triton X-100 solution for 20 min. The cells were then blocked with 5% BSA for 30 min to prevent non-specific binding. Subsequently, the cells were incubated with the primary antibody (diluted 1:200) and Alexa Fluor-conjugated secondary antibody, including a negative control (secondary antibody only). A solution of 200 µL PBS containing 1–5 µL of fluorescently labeled phalloidin (CoraLite^®^ 594, Proteintech, Wuhan, China) was added to each well and incubated at room temperature for 20 min. The nuclei were then stained with DAPI (diluted 1:1000) for 15 min. Finally, the cells were observed and analyzed using a confocal laser scanning microscope (Zeiss LSM 880, Nikon Corporation, Tokyo, Japan).

### 2.11. Chromatin Immunoprecipitation (ChIP) Assay

ChIP was performed using a ChIP assay kit (P2078; Beyotime Biotechnology, Shanghai, China) according to the manufacturer’s instructions and published protocols [17]. In brief, 3 × 10^6^ PANC-1 cells were cross-linked, lysed, and sonicated to generate sheared chromatin, and 10% of the sample was reserved as input. Chromatin was immunoprecipitated with 4 μg anti-E2F1 antibody (Proteintech, Wuhan, China, 66515-1-Ig) or normal rabbit IgG. DNA recovered from ChIP and input fractions were quantified by qPCR (primers specific for the WDHD1 promoter (left primer: 5′-CACCACTTCCGTGTTCAGCT-3′; right primer: 5′-GCCCTCCGCGTGGAAATTAG-3′)). Primer positions across the WDHD1 promoter are detailed in Supplementary Data S1. Enrichment was calculated as fold change relative to the IgG negative control.

### 2.12. Luciferase Reporter Assay

Luciferase reporter assays were performed as follows [17]. PANC-1 cells (approximately 2 × 10^6^) were seeded and cultured overnight to reach 70–90% confluence. For each well, a 500 µL transfection mixture was prepared comprising 250 µL Opti-MEM, 1000 ng firefly luciferase reporter plasmid, 100 ng Renilla luciferase control plasmid, and 2 µL P3000 reagent. In a separate tube, 250 µL Opti-MEM was mixed with 2 µL Lipofectamine 3000. The two mixtures were combined, incubated at room temperature for 10 min, and added dropwise to the cells. Cells were maintained at 37 °C in 5% CO_2_ for 24–48 h. Relative luciferase activity was quantified using a luciferase assay kit (RG088S; Beyotime Biotechnology, Shanghai, China), with firefly luminescence normalized to Renilla activity.

### 2.13. Clinical Samples and Data Collection

Pancreatic cancer RNA-seq data were obtained from the Cancer Genome Atlas (TCGA) database (https://www.cancer.gov/tcga, accessed 30 September 2022). RNA-seq data for pancreatic adenocarcinoma (TCGA-PAAD) tumors and normal pancreas tissues (GTEx) were queried via GEPIA2 (http://gepia2.cancer-pku.cn), which integrates TCGA and GTEx expression profiles processed uniformly through the UCSC Xena pipeline (values as log_2_(TPM + 1)). In all primary tumor-versus-normal analyses, “normal” controls were defined as GTEx normal pancreas samples integrated by GEPIA2. TCGA adjacent non-tumor tissues were used only for sensitivity checks due to their limited availability in PAAD and were not considered primary controls. Differential expression was assessed using GEPIA’s default one-way ANOVA framework on log_2_(TPM + 1) values. When multiple genes were evaluated, *p*-values were adjusted using the Benjamini–Hochberg procedure to control the false-discovery rate, and adjusted q-values are reported where applicable. Survival analyses were performed on TCGA tumor cases only. Patients were dichotomized by the median expression (unless otherwise specified), Kaplan–Meier curves were compared by two-sided log-rank tests, and hazard ratios with 95% confidence intervals were estimated using Cox proportional hazard models. For survival screens involving multiple genes, BH-FDR correction was applied. For a priori, hypothesis-driven single-gene tests, nominal *p*-values are presented as descriptive statistics. All primary pancreatic cancer and adjacent non-cancerous tissue samples were obtained through surgical resection from patients at West China Hospital, Sichuan University (Chengdu, China), between 2020 and 2021. Immediately following resection, the samples were preserved in liquid nitrogen. All sample processing procedures were conducted on ice to ensure sample integrity. Informed consent was obtained from all patients in accordance with institutional policies. This study was conducted in compliance with the principles of the Declaration of Helsinki and was approved by the Ethics Committee of West China Hospital, Sichuan University (approval number 2021review [1189]).

### 2.14. Immunohistochemistry (IHC)

IHC was evaluated by two board-certified pathologists blinded to clinical data and outcomes [18,19]. Staining was quantified using a standardized H score (0–300), calculated as intensity (0–3) × percentage of positive tumor nuclei. Discrepancies were resolved by consensus, and interobserver agreement was assessed. Staining runs were standardized (identical antigen retrieval, antibody dilution, incubation, and DAB development times) with internal controls and negative controls included for each batch. We also specify the predefined cutoffs used for downstream analyses.

### 2.15. Subcutaneous Tumor Xenograft Assay

All animal experimental protocols were approved by the Animal Ethics and Treatment Committee of Sichuan University (Chengdu, China) and adhered to the NIH *Guide for the Care and Use of Laboratory Animals*. The xenograft model was established using NOD (NOD/ShiLtJGpt-Prkdcem26Cd52Il2rgem26Cd22/Gpt) severely immunodeficient female mice (purchased from Jiangsu GemPharmatech Co., Ltd., Nanjing, China; 20 g, 4–6-week-old females), randomly assigned to groups (*n* = 5 per group). Sample size justification: Prior to the main study, we conducted a pilot under identical conditions (cell line, inoculum, site, timeline), which showed a high engraftment rate (≥85%) and a 20–30% coefficient of variation in tumor volumes at day 21. Using these pilot estimates, an a priori power analysis (two-sided α = 0.05) targeting a ~45–50% reduction in endpoint tumor volume (or growth-curve AUC) indicated 80–85% power with *n* = 5 per group. The observed take rate in the current cohort matched the pilot. PANC-1 cells, either transfected with an empty vector or a WDHD1 knockdown vector (1 × 10^7^ cells per mouse), were subcutaneously injected into the right flank of the mice. Beginning on day 7 after cell inoculation, the longest diameter (length) and the shortest diameter (width) of the tumors were measured using calipers. Measurements were performed by the same researcher, who was blinded to the group assignments. All procedures were conducted in the experimental operation room of an SPF-grade animal facility. Tumor volume was quantified with the formula: volume = (width^2^ × length)/2. Primary outcome measure: tumor volume. At the study endpoint, animals were anesthetized with tribromoethanol (15 mg/mL, intraperitoneally), and tumor tissues were collected for immunoblotting. Statistical methods are described in the Statistical Analysis section.

The inclusion criteria for this study were animals in good health, weighing 20 g, 4- to 6-week-old female mice, and those that successfully completed the model construction. The exclusion criteria were animals that died accidentally during the experiment, failed to develop tumors, or showed severe infections. During data analysis, the average of all technical replicates was considered a single independent data point, and any data points falling outside the mean ± 2 standard deviations were regarded as outliers and excluded. These criteria were predefined (a priori). All data from this study were included in the analysis without any exclusions.

Strategies used to minimize potential confounders include maintaining consistent order of treatments and measurements between the control and experimental groups, as well as ensuring the animal/cage location is consistent between the control and experimental groups. During the allocation phase: Group allocation was performed by Xiaojuan Yang, who was aware of the assignments. This was necessary to ensure that group injections were conducted according to a random-number table. During the experiment: Zhiwei Zhang, responsible for daily animal care, was blinded to the group assignments. During the outcome assessment: Shuangjuan Lv, the researcher assessing the outcomes, was blinded to the group information. To ensure objectivity, animals were renumbered, and measurements were taken by personnel who were unaware of the group assignments. During the data analysis: Shuangjuan Lv, the researcher conducting the data analysis, was blinded to the group information. Data analysis was performed using anonymized data files, with groups labeled “Group A” and “Group B” rather than “Control Group” and “Experimental Group.”

### 2.16. Weighted Gene Co-Expression Network Analysis (WGCNA)

Hub gene screening and co-expression of gene pair detection were carried out using the R package WGCNA [20]. Briefly, WGCNA was performed using the WGCNA package on normalized RNA-seq data from 178 TCGA pancreatic cancer samples. After quality control, low-variance genes were filtered and ~20,000 most variable genes were retained. To satisfy the scale-free topology criterion, we evaluated candidate soft-thresholding powers with pickSoftThreshold and selected *β* = 12 as the minimal power yielding a high scale-free fit while maintaining reasonable mean connectivity. Module detection and module–trait correlations were confirmed to be stable across neighboring powers. A signed adjacency matrix (biweight mid-correlation) was computed and transformed to a topological overlap matrix (TOM). Genes were clustered by average linkage, modules were identified using dynamic tree cutting (followed by module merging), and module eigengenes were calculated [21,22]. Module–trait associations were estimated by biweight mid-correlation between module eigengenes and clinical traits with Benjamini–Hochberg correction. Hub genes were prioritized by intramodular connectivity and module membership, together with gene significance for the trait. Gene–gene co-expression networks were exported from TOM for visualization in Cytoscape (version 3.10.3, Cytoscape Consortium, San Diego, CA, USA).

### 2.17. Statistical Analysis

Statistical analyses in this study were performed using GraphPad Prism 8.0 software. All data are presented as means ± standard deviation (mean ± SD) from at least three independent experiments. Normality of data distribution was assessed using the Kolmogorov–Smirnov test, Anderson–Darling test, Jarque–Bera test, or Shapiro–Wilk test, and homoscedasticity was evaluated using the F–test, Brown–Forsythe test, or Bartlett’s test. Depending on the results of these tests, parametric analyses were conducted using one-way ANOVA followed by post hoc tests (e.g., Bonferroni, Tukey) or Student’s t-tests. For data sets that did not meet the assumptions for parametric tests, non-parametric tests were employed. All statistical tests were two-sided, with a *p*-value of <0.05 considered statistically significant, denoted as follows: * *p* < 0.05; ** *p* < 0.01; *** *p* < 0.001; and **** *p* < 0.0001.

## 3. Results

### 3.1. Screening for Target Genes

WGCNA was applied to RNA-seq data from 178 TCGA pancreatic cancer samples. We constructed a weighted co-expression network across ~20,000 genes, and to satisfy the scale-free topology criterion, systematically evaluated candidate soft-thresholding powers using pickSoftThreshold. A power of 12 was chosen as the minimal value achieving a high scale-free fit while maintaining reasonable connectivity, and module detection as well as module–trait correlations were stable across neighboring powers (see Methods). Using dynamic tree cutting with subsequent module merging, the 20,000 genes were partitioned into twelve co-expression modules (Supplementary Figure S1A,B). Module–trait relationships were then computed, and the pink module exhibited the strongest and most robust association with overall survival (Supplementary Figure S1C,D), suggesting that its member genes may cooperatively influence pancreatic cancer progression and outcomes. In line with recent recommendations emphasizing robust regulatory network context for prognostic modules and cross-cohort transcriptome integration for gene screening, we contextualized our analysis using Qian et al. and Tang et al. [21,22]. Within the pink module, WDHD1 showed high module membership and gene significance, and its prioritization remained stable under the above sensitivity assessments.

To identify potential therapeutic targets in pancreatic cancer, we further screened for key node genes within the pink gene module. The screening criteria included: (1) node gene expression levels significantly correlated with pancreatic cancer survival prognosis; (2) these genes exhibited significant differential expression between pancreatic cancer tissues and normal control tissues; and (3) priority was given to genes that have been less extensively studied in the field of oncology, especially those that have not yet been investigated in pancreatic cancer. Then, 32 node genes emerged as candidates (Supplementary Figure S1E). After a comprehensive evaluation and literature review, we ultimately selected the *WDHD1* gene for further study. WDHD1 met all specified criteria, demonstrating significant prognostic associations and marked differential expression between pancreatic cancer and normal tissues. Additionally, research on *WDHD1* in the context of pancreatic cancer has been relatively limited compared to other known cancer-related genes.

### 3.2. High Expression of WDHD1 in Pancreatic Cancer

**Figure 1:**
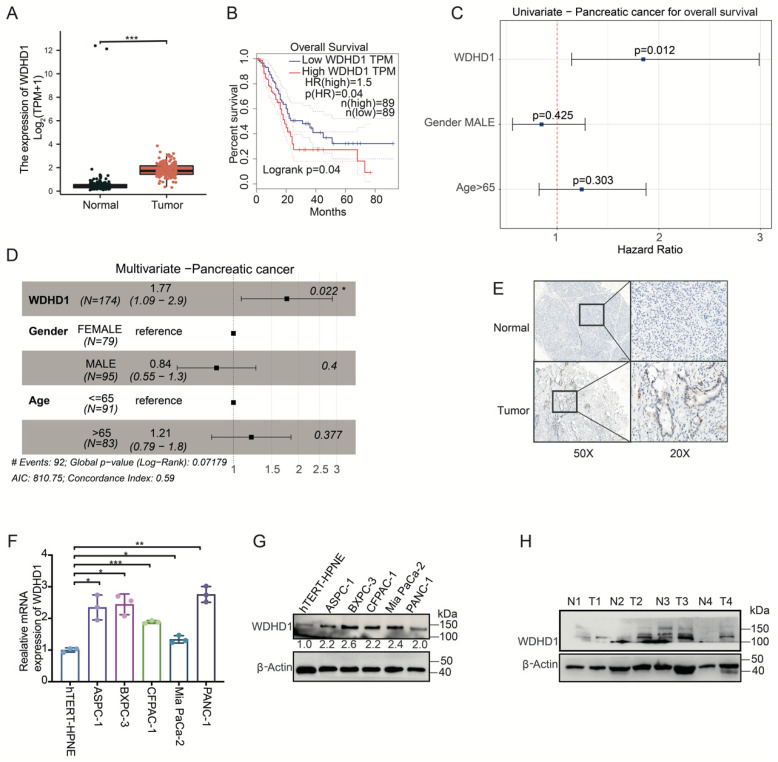
High expression of WDHD1 in pancreatic cancer patients. (**A**) Comparison of WDHD1 expression levels between normal and pancreatic cancer tumor samples shows significantly higher expression in tumors. *** *p* < 0.001 (Student’s *t*-test). (**B**) Survival analysis indicates that higher *WDHD1* gene expression correlates with poorer prognosis in pancreatic cancer patients (*n* = 89 for high expression of WDHD1, *n* = 89 for low expression of WDHD1). (**C**) Univariate survival association of WDHD1 in TCGA–pancreatic adenocarcinoma. (**D**) Multivariate Cox model adjusting for clinicopathologic covariates. * *p* < 0.05 (Student’s *t*-test). (**E**) Immunohistochemical results demonstrate that WDHD1 is highly expressed in pancreatic cancer tissues, particularly in the nuclear regions, compared to adjacent normal tissues. (**F**) RNA expression levels of WDHD1 in various pancreatic cancer cell lines show elevated expression compared to normal pancreatic cell lines (*n* = 3 independent biological replicates). Summary statistics for each experimental group reported as means ± SD. Statistical significance: * *p* < 0.05, ** *p* < 0.01, *** *p* < 0.001 (Student’s *t*-test). (**G**) Protein expression levels of WDHD1 in pancreatic cancer cell lines indicate significant upregulation compared to non-cancerous cell lines. β-actin served as a loading control. Representative images were selected from three independent experiments. (**H**) Western blot results confirm that WDHD1 expression is markedly higher in pancreatic cancer tissues compared to adjacent non-tumor tissues. β-actin served as a loading control. Representative images were selected from three independent experiments (*n* = 4 for tumor samples, *n* = 4 for and adjacent samples). Due to the nature of archived clinical samples, some protein degradation may occur, and the bands shown represent the specific signal for WDHD1. The unedited blots can be found in Supplementary File S1.

The GEPIA database was used to analyze WDHD1 expression in pancreatic cancer and its relationship with patient outcomes [23]. Figure 1A illustrates a significant increase in WDHD1 expression in pancreatic cancer tissues, indicating its potential role in tumorigenesis. Additionally, pancreatic cancer patients with higher *WDHD1* gene expression levels tended to have poorer prognoses (Figure 1B). To address potential confounding in survival analyses, we reanalyzed the TCGA–pancreatic adenocarcinoma cohort using Cox proportional hazard models. On univariate analysis, higher WDHD1 expression was associated with shorter overall survival (Figure 1C). Critically, in multivariate models adjusting for key clinicopathologic factors, WDHD1 remained an independent prognostic factor (Figure 1D). This finding was consistent whether WDHD1 expression was modeled as a continuous variable or dichotomized at the median.

To further investigate whether WDHD1 is overexpressed in clinical samples, we collected tissue samples from a cohort of pancreatic cancer patients and used IHC to detect WDHD1 expression (Figure 1E). Normal pancreatic tissues had very low WDHD1 expression, but pancreatic tumor tissues showed high WDHD1 expression, particularly in the nucleus. The expression of WDHD1 in different pancreatic cell lines was assessed by Western blot and qPCR (Figure 1F,G). WDHD1 expression was significantly higher in multiple pancreatic tumor cell lines compared to non-cancerous HPNE cells. WDHD1 expression was notably elevated in tumor tissues compared to adjacent non-tumor tissues (Figure 1H). The patients’ baseline clinicopathologic characteristics were balanced. Please refer to our previously published paper for details [24].

### 3.3. Effect of WDHD1 on Proliferation of Pancreatic Cancer Cells

As shown in Figure 1G, WDHD1 expression varies across cell lines. We selected PANC-1 (*KRAS-G12D*) and CFPAC-1 (*KRAS-G12V*) for loss-of-function studies because these alleles represent the most prevalent *KRAS* genotypes in pancreatic ductal adenocarcinoma, whereas BxPC-3 is wild-type *KRAS* and therefore less representative of canonical PDAC genetics. In addition, pilot transfection/transduction in MiaPaCa-2 yielded variable efficiencies and unstable phenotypes, precluding reproducible analyses. By contrast, PANC-1 and CFPAC-1 supported robust genetic manipulation and consistent readouts. Notably, baseline WDHD1 abundance is not necessarily a surrogate for functional dependency. Our aim was to test essentiality in genetically representative and experimentally tractable models.

**Figure 2:**
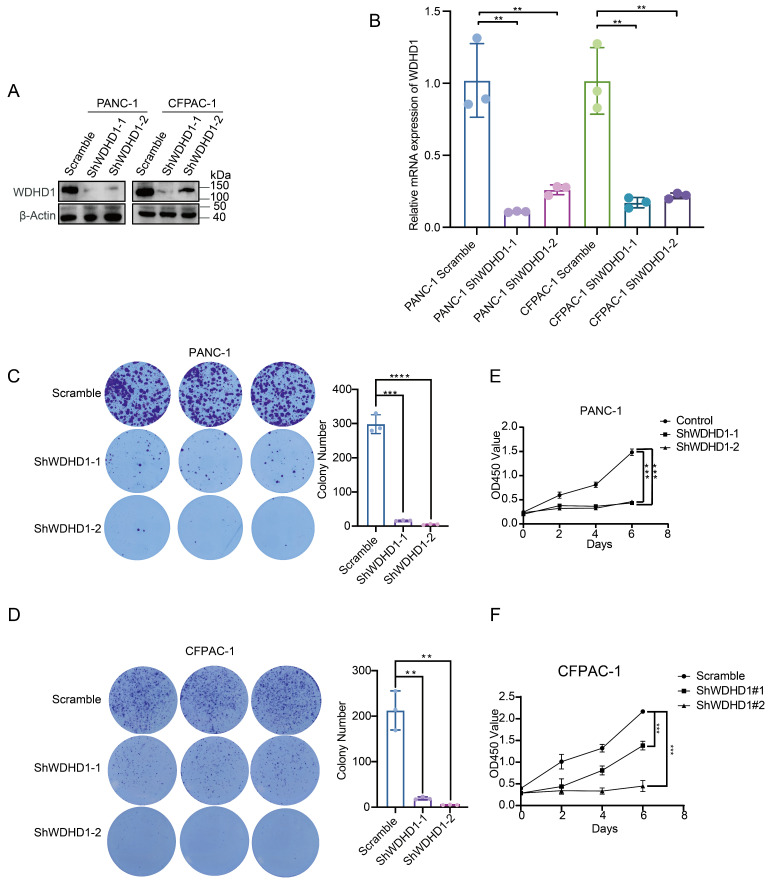
Downregulation of WDHD1 inhibits the proliferation of pancreatic cancer cells. (**A**) Western blot verification of the protein knockdown level of WDHD1 in the pancreatic cancer cell line. β-actin served as a loading control; Representative images were selected from three independent experiments. (**B**) qPCR verification of the RNA knockdown level of WDHD1 in pancreatic cancer cell lines (*n* = 3 independent biological replicates). Summary statistics for each experimental group reported as means ± SD. Statistical significance: ** *p* < 0.01 (Student’s *t*-test). (**C**,**D**) Colony formation assays show a decrease in the number of clones in pancreatic cancer cell lines after WDHD1 knockdown. Representative images were selected from three independent experiments. Summary statistics for each experimental group are reported as means ± SD. Statistical significance: ** *p* < 0.01, *** *p* < 0.001, **** *p* < 0.0001 (Student’s *t*-test). (**E**,**F**) CCK-8 cell proliferation assays demonstrate that WDHD1 knockdown inhibits the proliferation rate of pancreatic cancer cells. *n* = 3 (independent biological replicates). Summary statistics for each experimental group are reported as means ± SD; Statistical significance: *** *p* < 0.001 (Student’s *t*-test). The unedited blots can be found in Supplementary File S1.

Building on prior research suggesting WDHD1’s involvement in DNA replication [25], we hypothesized its significant role in pancreatic cancer cell proliferation. To test this hypothesis, we established a pancreatic cancer cell model with *WDHD1* knockdown gene expression and validated the knockdown at both RNA and protein levels (Figure 2A,B). Cells with WDHD1 knockdown exhibited a significant reduction in colonies (Figure 2C,D). As shown in Figure 2E,F, the knockdown of WDHD1 significantly reduced the proliferation rate of pancreatic cancer cells. Similarly, overexpression of WDHD1 promoted the proliferation of pancreatic cancer cells (Supplementary Figure S2).

**Figure 3:**
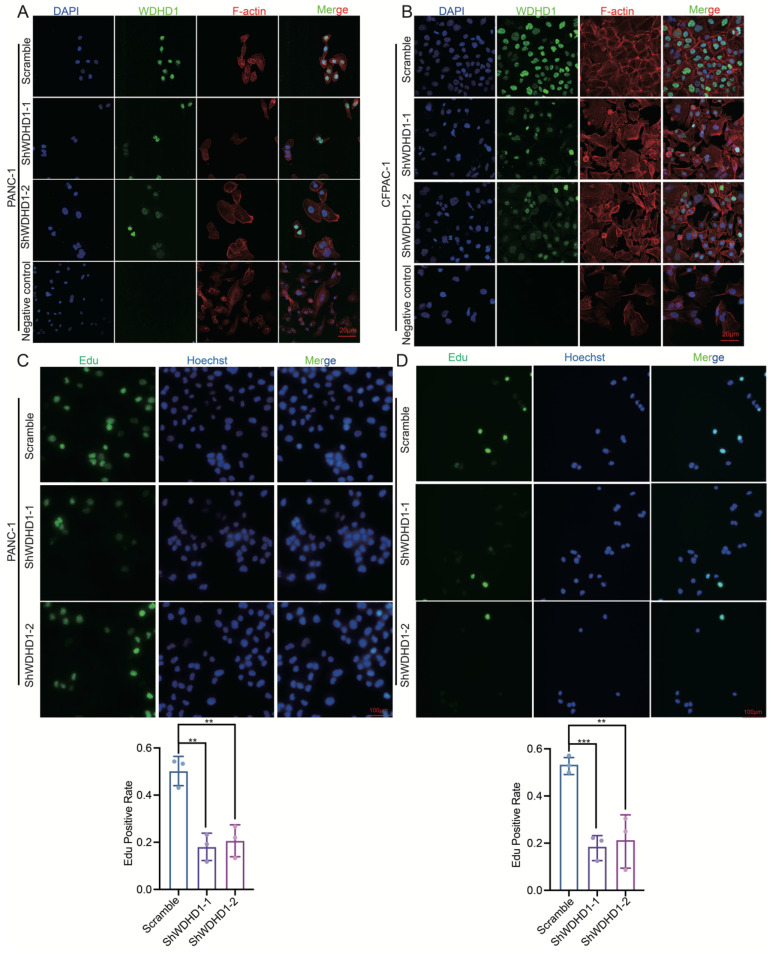
Immunofluorescence and EdU experimental results after knocking down WDHD1. (**A**) Knockdown of WDHD1 immunofluorescence in PANC-1 cell line, where WDHD1 is mainly located in the nucleus. Representative images were selected from three independent experiments; Scale bar: 20 μm. “Secondary antibody alone” was used as the negative control staining. Blue: DAPI; Green: WDHD1; Red: F-actin. (**B**) Knockdown of WDHD1 immunofluorescence in CFPAC. Representative images were selected from three independent experiments. Scale bar: 20 μm. “Secondary antibody alone” was used as the negative control staining. Blue: DAPI; Green: WDHD1; Red: F-actin. (**C**,**D**) EdU detection of WDHD1 knockdown in pancreatic cancer cell lines showed that WDHD1 knockdown could inhibit DNA replication. Green: Edu; Blue: Hoechst. *n* = 3 (independent biological replicates). Scale bar: 100 μm; Representative images were selected from three independent experiments. Summary statistics for each experimental group reported as means ± SD. Statistical significance: ** *p* < 0.01, *** *p* < 0.001 (Student’s *t*-test).

Immunofluorescence validated WDHD1 knockdown efficiency and its primary nuclear localization in pancreatic cancer cell lines, suggesting its main functions are nuclear (Figure 3A,B). The EdU assay results after *WDHD1* gene knockdown highlight its essential role in DNA replication. WDHD1 knockdown in pancreatic cancer cell lines significantly reduced the proportion of EdU-positive cells (Figure 3C,D).

### 3.4. Effect of WDHD1 on Pancreatic Cancer Cell Cycle and Apoptosis

**Figure 4:**
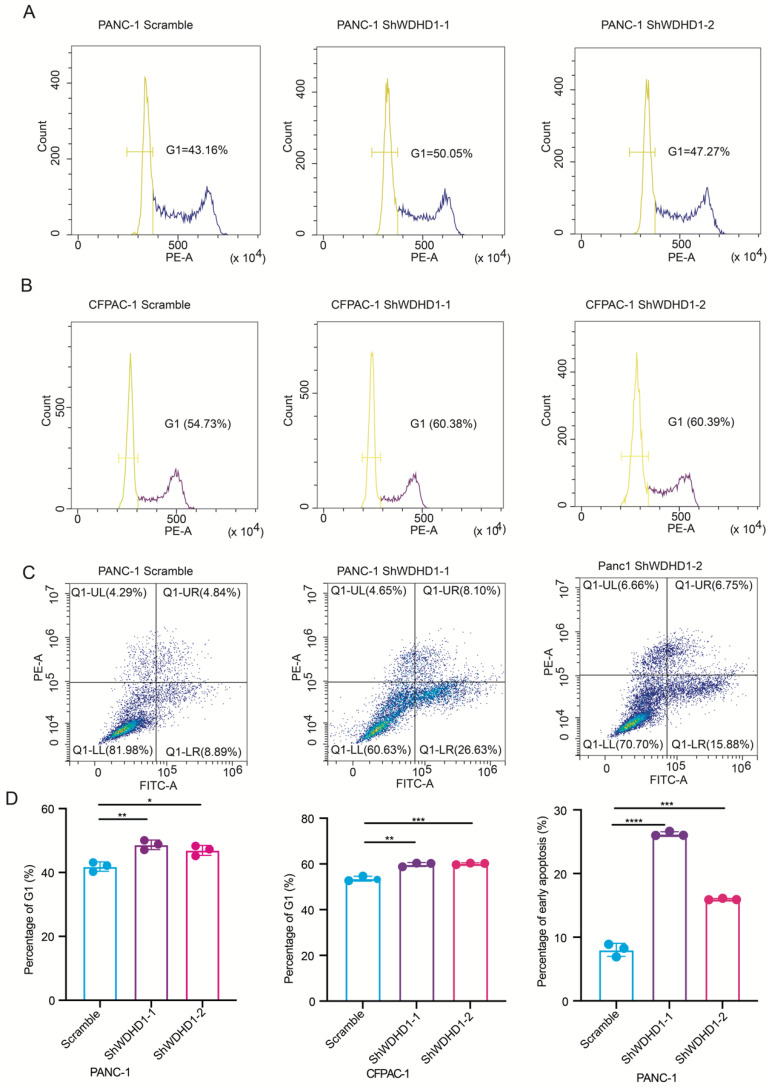
The effect of WDHD1 knockdown on cell cycle and apoptosis of pancreatic cancer. (**A**,**B**) Flow cytometry analysis showing the impact of WDHD1 knockdown on cell-cycle distribution. (**C**) Flow cytometry analysis demonstrating the effect of WDHD1 knockdown on apoptosis. (**D**) Quantification of G1-phase cells in PANC-1 and CFPAC-1 from (**A**,**B**), and early apoptosis rates in PANC-1 from (**C**) (*n* = 3 independent biological replicates). Summary statistics for each experimental group presented as means ± SD. Statistical significance was determined by Student’s *t*-test (* *p* < 0.05, ** *p* < 0.01, *** *p* < 0.001, **** *p* < 0.0001).

A significant increase in pancreatic cancer cells in the G1 phase was observed in the knockdown group (Figure 4A,B). This result suggests that suppression of WDHD1 leads to a notable arrest in the G1 phase, referred to as G1-phase blockade. We utilized flow cytometry to examine the apoptotic status of pancreatic cancer cells after knocking down WDHD1 expression (Figure 4C). Overall, WDHD1 knockdown in pancreatic cancer cells significantly increased G1-phase arrest and the number of early apoptotic cells compared with controls (Figure 4D).

### 3.5. The E2F1–WDHD1 Axis Drives Pancreatic Cancer Progression by Activating the CDK4–Cyclin D1 Complex

To explore WDHD1 regulatory mechanisms, we screened and identified the 200 genes most correlated with WDHD1 from the GEPIA database [23]. We then performed an intersection analysis using the list of predicted transcription factors from the GeneHancer database with these two datasets (Supplementary Figure S3A) to identify potential key transcription factors that directly regulate WDHD1 expression.

Through data mining and bioinformatic analysis, we identified six potential transcription factors for WDHD1: E2F1, POU5F1, TRIM28, BRCA1, FOXM1, and RAD51. Given that previous research on cervical cancer has shown WDHD1 to be closely associated with cell-cycle regulation [9], our study also suggests that WDHD1 influences cell-cycle progression in pancreatic cancer. Additionally, we found that the expression levels of WDHD1 in pancreatic cancer are significantly positively correlated with E2F1 (Supplementary Figure S3B). Therefore, we hypothesize that E2F1 may directly regulate WDHD1 transcription. The CCK-8 cell proliferation and colony formation assays revealed that E2F1 overexpression significantly boosts cell proliferation, while E2F1 knockdown suppresses it (Supplementary Figure S3C–F). Given that E2F1 and WDHD1 exhibit similar phenotypic characteristics in cell proliferation effects, this suggests that WDHD1 may act as a downstream molecule involved in E2F1-mediated cell proliferation regulation.

**Figure 5:**
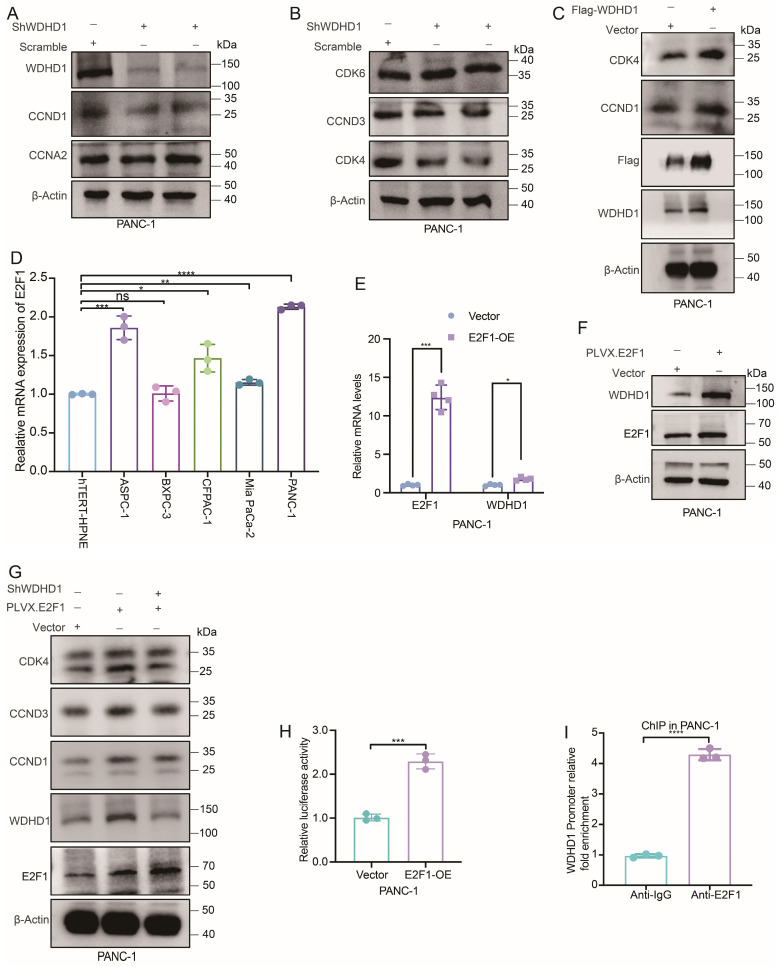
The E2F1–WDHD1 axis promotes malignant phenotypes in pancreatic cancer by upregulating cyclin D1. (**A**,**B**) Expression of cell cycle-related proteins after knocking down WDHD1. β-actin served as a loading control; Representative images were selected from three independent experiments. (**C**) Expression of CDK4 and cyclin D proteins after overexpression of WDHD1. β-actin served as a loading control. Representative images were selected from three independent experiments. (**D**) RNA expression levels of E2F1 in various pancreatic cancer cell lines show elevated expression compared to normal pancreatic cell lines (*n* = 3 independent biological replicates). Summary statistics for each experimental group reported as means ± SD. Statistical significance: * *p* < 0.05, ** *p* < 0.01, *** *p* < 0.001, **** *p* < 0.0001 (Student’s *t*-test), ns, non-significant. (**E**,**F**) Overexpression of E2F1 can increase the expression of *WDHD1* at both the RNA and protein levels (*n* = 4 independent biological replicates). Summary statistics for each experimental group reported as means ± SD. Statistical significance: * *p* < 0.05, *** *p* < 0.001 (Student’s *t*-test). β-actin served as a loading control. Representative images were selected from three independent experiments. (**G**) The impact of WDHD1 knockdown after overexpression of E2F1 on cyclin D1, cyclin D3, and CDK4 at protein level. β-actin served as a loading control. Representative images were selected from three independent experiments. (**H**) E2F1 activates the WDHD1 promoter. E2F1 overexpression significantly increased the activity of a luciferase reporter driven by the full-length WDHD1 promoter relative to the vector control, consistent with direct transcriptional activation of WDHD1 by E2F1 (*n* = 3 independent biological replicates). Summary statistics for each experimental group reported as means ± SD; Statistical significance: *** *p* < 0.001 (Student’s *t*-test). (**I**) ChIP–qPCR shows enrichment of the *WDHD1* promoter in anti-E2F1 immunoprecipitates relative to IgG control (*n* = 3 independent biological replicates). Summary statistics for each experimental group reported as means ± SD; Statistical significance: **** *p* < 0.0001 (Student’s *t*-test). The unedited blots can be found in Supplementary File S1.

To assess the impact of WDHD1 on cell-cycle effectors, we profiled CDK4 and cyclin D1 by immunoblotting in pancreatic cancer cells following WDHD1 depletion (Figure 5A,B). WDHD1 knockdown was accompanied by a marked reduction in CDK4 and cyclin D1 protein abundance, consistent with restraint of the G1–S transition. Conversely, ectopic WDHD1 increased CDK4 and cyclin D1 protein levels (Figure 5C). At the transcript level, WDHD1 depletion lowered CDK4 and E2F1 mRNAs, whereas CCND1 mRNA increased (Supplementary Figure S4A), indicating discordance between mRNA and protein that is compatible with checkpoint activation and post-transcriptional control of cyclin D1. Taken together, these observations indicate that loss of WDHD1 is associated with suppression of the cyclin D1–CDK4 axis; however, they do not establish direct regulation of CDK4 or CCND1 by WDHD1. Moreover, literature and database sources (https://www.uniprot.org/uniprotkb/P06400/entry, accessed on 11 November 2025) suggest that when interacting with CDK4, members of the cyclin D family (such as cyclin D1/D3) can phosphorylate the retinoblastoma (Rb) tumor suppressor protein, transforming Rb from an inhibitory state to an active state, which in turn releases the E2F1 transcription factor [26,27], raising the possibility of a positive feedback loop involving E2F1–WDHD1–CDK4/cyclin D1–E2F1.

To verify that E2F1 is an upstream regulatory factor of WDHD1, we analyzed the mRNA expression of E2F1 in normal pancreatic epithelial cells (hTERT-HPNE) and in pancreatic cancer cell lines (ASPC-1, BXPC-3, CFPAC-1, Mia PaCa-2, and PANC-1). Consistently with WDHD1 expression, we found that E2F1 is significantly overexpressed in ASPC-1, CFPAC-1, Mia PaCa-2, and PANC-1 cells, and shows a trend of high expression in BXPC-3 cells (Figure 5D). Furthermore, we found that overexpressing E2F1 can elevate the expression of WDHD1 at both the protein and RNA levels (Figure 5E,F). The above results suggest that E2F1 promotes the expression of WDHD1 at both mRNA and protein levels, while WDHD1 enhances the expression of CDK4 and cyclin D1 proteins, thereby promoting the progression of pancreatic cancer.

To validate that the E2F1–WDHD1 axis regulates CDK4, we performed rescue experiments. The results demonstrated that WDHD1 knockdown mitigated the upregulation of cyclin D1 and CDK4 protein expression induced by E2F1 overexpression (Figure 5G). Additionally, the results shown in Supplementary Figure S4B suggest that WDHD1 may influence CDK4 protein levels independently of mRNA. These findings suggest that E2F1 activates WDHD1, which subsequently enhances the expression of cyclin D1 and CDK4. When WDHD1 is knocked down, the ability of E2F1 to promote the expression of cyclin D1 and CDK4 is diminished, confirming that WDHD1 is a critical mediator in this pathway.

Finally, we interrogated the Cistrome data browser (https://db3.cistrome.org/browser/, accessed on 10 November 2025), which indicated E2F1 occupancy at the *WDHD1* promoter. A full-length *WDHD1* promoter–luciferase reporter showed a significant increase in activity upon E2F1 overexpression, consistent with direct transcriptional activation of *WDHD1* by E2F1 (Figure 5H). ChIP–qPCR in PANC-1 cells demonstrated significant enrichment of *WDHD1* promoter fragments in anti-E2F1 immunoprecipitates relative to IgG control, indicating direct binding of E2F1 to the *WDHD1* promoter and supporting *WDHD1* as a transcriptional target of E2F1 (Figure 5I). These data support a regulatory link between E2F1 and WDHD1 expression, in line with the changes in cell-cycle regulators observed elsewhere in Figure 5.

### 3.6. WDHD1 Promotes the Proliferation of Pancreatic Cancer Cells In Vivo

**Figure 6:**
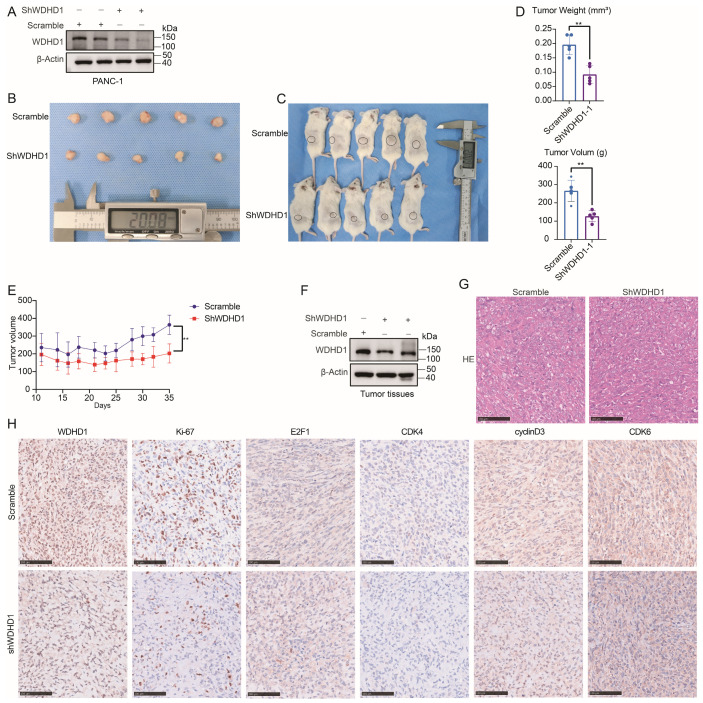
Effect of WDHD1 knockdown on tumorigenesis of pancreatic cancer cells *in vivo*. (**A**) Validation of WDHD1 knockdown in PANC-1 cells before subcutaneous injection into mice. β-actin served as a loading control. Representative images were selected from three independent experiments. (**B**,**C**) Comparison of subcutaneous tumor tissue volume between the WDHD1 knockdown group and the control group in mice. (**D**) Statistical analysis of tumor volume between the two groups, showing that the knockdown group had a significantly smaller tumor volume (*n* = 5, “*n*” defines the number of mice in each group). Summary statistics for each experimental group reported as means ± SD. Statistical significance: ** *p* < 0.01 (Student’s *t*-test). (**E**) Growth curves of the two groups of tumors, indicating that the subcutaneous tumor volume in the knockdown group increased more slowly (*n* = 5, “*n*” defines the number of mice in each group). Summary statistics for each experimental group reported as means ± SD. Statistical significance: ** *p* < 0.01 (Student’s *t*-test). (**F**) Western blot detection of WDHD1 protein expression in subcutaneous tumor tissue. β-actin served as a loading control. Representative images were selected from three independent experiments. (**G**,**H**) Representative images and quantification of WDHD1, Ki-67, E2F1, CDK4, CDK6, and cyclin D3 immunohistochemical detection in murine subcutaneous tumor tissues. Scale bar: 100 μm. The unedited blots can be found in Supplementary File S1.

To partially validate our *in vitro* findings on the role of WDHD1 in the proliferation of pancreatic cancer cells, we established subcutaneous xenograft models in mice using PANC-1 cells with WDHD1 knocked down (Figure 6A). During the experiment, the volume of the xenografts was measured every two days using calipers to record changes in tumor size and plot tumor growth curves. After 35 days of observation, the experimental mice were euthanized by cervical dislocation, and the subcutaneous tumor tissues were excised (Figure 6B,C).

Statistical analysis revealed that the *WDHD1* gene knockdown group exhibited a significantly reduced tumor growth rate, as well as a notable decrease in both tumor size and weight (Figure 6D,E). This finding aligns with *in vitro* experimental conclusions, reinforcing WDHD1’s role in promoting pancreatic cancer cell proliferation.

Western blot analysis indicated a significant reduction in WDHD1 protein expression in transplanted tumor tissues of WDHD1 knockdown mice (Figure 6F).

H&E staining confirmed that the subcutaneous tumors displayed histological features consistent with pancreatic adenocarcinoma (Figure 6G). Immunohistochemistry revealed that WDHD1 knockdown significantly reduced the proliferation marker Ki-67 and decreased CDK4, CDK6, and cyclin D3 levels, whereas E2F1 expression was unchanged (Figure 6H). As WDHD1 knockdown in Figure 4C markedly increased apoptosis, we examined its *in vivo* effects on apoptosis-associated proteins. IHC of mouse subcutaneous tumors revealed that WDHD1 knockdown significantly increased caspase 3, cleaved caspase 3 (Asp175), and caspase 9 (Supplementary Figure S5), consistent with enhanced apoptotic signaling.

**Figure 7:**
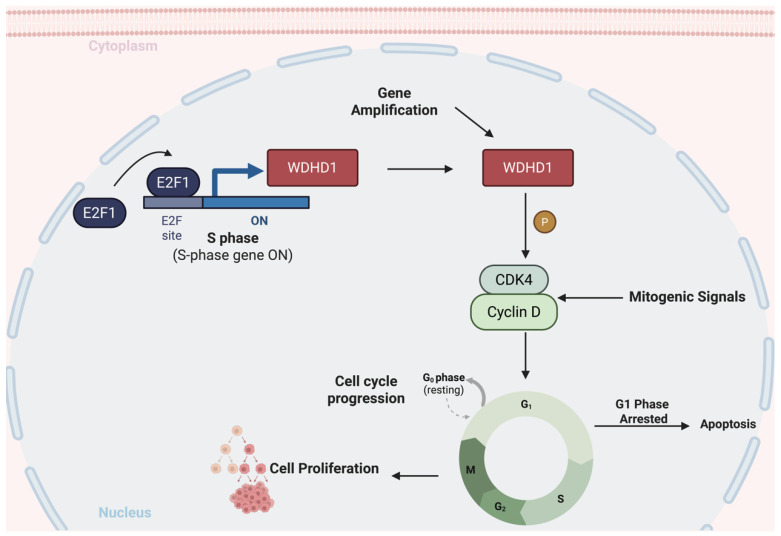
Graphical model of the E2F1–WDHD1 axis regulating cyclin D1 to promote pancreatic cancer malignancy. E2F1 transcriptionally activates WDHD1, increasing WDHD1 protein abundance. Elevated WDHD1, in turn, enhances the expression of key G1 regulators, notably cyclin D1 and CDK4, promoting formation and activation of the cyclin D1–CDK4 complex.

In summary, E2F1 promotes the transcription of WDHD1, thereby increasing its protein levels. In turn, WDHD1 enhances the expression of key cell-cycle proteins, such as cyclin D and CDK4, thereby promoting cell-cycle progression and the advancement of pancreatic cancer (Figure 7).

E2F1 can directly regulate the transcription of WDHD1, which in turn promotes the expression of cell-cycle regulatory proteins CDK4 and cyclin D. This cascade facilitates cell-cycle progression and contributes to the malignant phenotype of pancreatic cancer.

## 4. Discussion

Tumor initiation and progression are rooted in genetic alterations and dysregulated proliferation, frequently accompanied by aberrant cell-cycle progression. Because the cell cycle is tightly controlled by multiple regulators, its disruption enables unchecked proliferation and drives tumorigenesis. For example, the development of pancreatic tumors typically starts with a series of genetic mutations within pancreatic cells, including those in *KRAS*, *TP53*, and *CDKN2A* [1,28]. In this study, we found that E2F1 drives the transcription of WDHD1, leading to elevated protein levels. Consequently, WDHD1 upregulates the expression of essential cell-cycle proteins, including cyclin D and CDK4. This mechanism persistently drove cell-cycle progression, leading to the characteristic uncontrolled growth in pancreatic cancer cells.

Recent research has focused on treatments targeting *KRAS* gene mutations, with drugs like the KRAS-G12D inhibitor HRS-4642 demonstrating promising activity in clinical trials, thereby providing new therapeutic opportunities for pancreatic cancer patients with *KRAS* mutations [29]. However, the high rate of drug resistance and the low prevalence of targetable genomic alterations in pancreatic cancer underscore the need to discover new therapeutic targets and develop effective therapies.

*WDHD1* is overexpressed in various malignant tumor cells and plays a role in DNA replication [9,30] and DNA damage repair [8]. In this study, we observed that WDHD1 is overexpressed in pancreatic cancer and promotes the proliferation of pancreatic cancer cells. Furthermore, knockdown of WDHD1 elevated the proportion of cells in the G1 phase, suggesting that WDHD1 facilitates the G1–S transition in these cells. Furthermore, WDHD1 knockdown not only impedes cell-cycle progression but also triggers apoptotic mechanisms. This indicates that inhibiting WDHD1 expression with relevant drugs could suppress the cell-cycle progression of pancreatic tumor cells. These findings provide a theoretical basis for developing therapeutic strategies targeting WDHD1 and its associated signaling pathways in the future.

Mechanistically, although our data do not demonstrate direct transcriptional control of CCND1 or CDK4 by WDHD1, they are consistent with a model in which loss of the replication factor WDHD1 induces replication stress and checkpoint activation, leading to RB hypophosphorylation, reduced E2F output, and functional repression of the cyclin D1–CDK4 complex. In line with an E2F-driven circuit, E2F1 binds and activates the *WDHD1* promoter, positioning WDHD1 within a feed-forward proliferative pathway: E2F1 upregulates WDHD1 to support DNA replication, whereas WDHD1 loss dampens E2F activity and G1/S entry. The CDK–RB–E2F pathway integrates mitogenic signals to govern the G1–S transition, and CDK4/6 inhibitors are approved or in clinical trials [31]. Our results are compatible with WDHD1 indirectly sustaining E2F activity by maintaining cyclin D1 and CDK4 expression and function, thereby influencing downstream cell-cycle gene programs; however, definitive mechanistic validation will require further experiments.

Importantly, Chang et al. showed that the cell-autonomous E2F1/4–pRB/RBL2 axis is perturbed by *KRAS* mutations in ductal cells [32]. Among the pancreatic cancer lines we examined, only BxPC-3 is wild-type *KRAS*. Whether its higher E2F1 levels reflect this context warrants investigation. Moreover, Li et al. underscore the importance of phosphorylation-driven cascades (e.g., METTL14–MYC) in pancreatic proliferation [33], while Sun et al. highlight how post-transcriptional regulation can produce discordance between mRNA and total protein levels and functional outcomes [34]. These insights emphasize that phospho-state and activity readouts are critical complements in total protein measurements. We explicitly note this limitation and outline planned validations: phospho-RB (Ser807/811) normalized to total RB, CDK2 activation (Thr160 phosphorylation), E2F target readouts, and interrogation of upstream signaling nodes such as MYC-related phosphorylation cascades. These future studies will provide direct mechanistic confirmation of G1–S pathway inhibition under *WDHD1* depletion, while our current functional data support reduced cell-cycle progression.

Furthermore, Tong et al. emphasize standardized IHC procedures and quantitative scoring (including H score) for PI3K/AKT pathway markers. Our blinded H-score protocol is concordant with these principles [19]. Canbey et al. highlight the necessity of blinded, standardized scoring in pancreatic cancer cohorts for PD-L1. While PD-L1 requires membranous scoring and WDHD1 is a nuclear protein, the underlying requirements for blinding, standardized thresholds, and batch control are shared and are now explicitly documented in our study [18]. Thus, their standardization frameworks do not contradict our nuclear scoring approach. Rather, they support the rigor of our methodology.

In this study, we directly demonstrate that WDHD1 promotes G1–S cell-cycle progression and cell survival in PDAC cells, and that WDHD1 knockdown induces G1 arrest and apoptosis accompanied by reduced CDK4/CDK6/cyclin D3, while E2F1 levels are largely unchanged. Beyond our data, recent reports have identified bazedoxifene (BZA) and the small molecule [(E)-5-(3,4-dichlorostyryl)benzo[c][1,2]oxaborol-1(3H)-ol] (CH3) as preclinical WDHD1 inhibitors [35]. CH3 engages the WD40 domain to disrupt WDHD1 trimerization and enhance binding to the E3 ligase CUL4B, promoting WDHD1 ubiquitination and proteasomal degradation—an approach aligned with targeted protein degradation strategies [11,25,35]. These findings are promising, but remain speculative in the context of PDAC: to our knowledge, BZA/CH3 has not been evaluated in PDAC *in vivo*, and key pharmacokinetic, selectivity, and safety data are needed before clinical translation.

Situating WDHD1 within current PDAC therapeutics, its role at the G1–S checkpoint and in DNA replication suggests potential intersections with established strategies. For example, WDHD1 inhibition might complement cell cycle–directed approaches (e.g., CDK4/6 inhibitors) or agents that exploit replication stress/DNA damage (e.g., gemcitabine, platinum, or ATR/CHK1 inhibitors), and could conceivably interact with KRAS-pathway blockade (MEK/ERK), given KRAS-driven E2F activation and replication stress in PDAC. These potential combinations are hypothesis-generating and require rigorous validation in orthotopic/PDX models and, ultimately, clinical studies.

We also acknowledge limitations relevant to translation: our conclusions are primarily supported by *in vitro* assays and a single-center cohort, and mechanistic mapping downstream of WDHD1 remains incomplete. Notably, higher-molecular-weight WDHD1 bands in uncropped immunoblots likely reflect post-translational modifications or stable complexes, and their identities and functions warrant further study. Future work will evaluate WDHD1 targeting *in vivo*, define its interaction with KRAS/cell-cycle/DDR pathways, and test rational combinations in genetically and microenvironmentally diverse PDAC models.

Additionally, a key limitation of this study is the lack of independent external validation in public cohorts (e.g., ICGC or GEO). Our transcriptomic analyses rely on the integrated TCGA/GTEx framework, and our protein/IHC findings derive from a single-center cohort. Suitable external datasets with matched WDHD1 measurements (particularly at the protein level), paired tumor–adjacent pancreas tissues, comprehensive clinicopathologic annotation, and long-term outcomes are currently scarce. In addition, cross-platform and batch heterogeneity across public datasets poses nontrivial harmonization challenges. While the concordance among our qPCR, Western blot, IHC, and functional assays provides internal consistency, this does not substitute for independent replication. We therefore acknowledge that generalizability should be interpreted with caution, and we plan to validate these findings in ICGC-PAAD and GEO series using standardized batch-correction pipelines, and where feasible to corroborate protein-level associations in CPTAC proteomic resources and multi-center prospective cohorts.

Finally, we acknowledge that shRNA-mediated knockdown can incur off-target effects that may not be captured without transcriptome-level auditing. Although we mitigated this by using two non-overlapping shRNAs that yielded concordant phenotypes and by performing rescue with an shRNA-resistant *WDHD1* cDNA, RNA-seq–based profiling was not included and remains a limitation. Consistently with Li et al.’s recommendations for pancreatic assays and Liu et al.’s TF-centric specificity framework [36,37], future work will incorporate: (i) RNA-seq to define an on-target WDHD1 signature and identify any off-target pathways, benchmarked against rescue; (ii) orthogonal perturbations (siRNA and CRISPRi) to reproduce key phenotypes; and (iii) target-proximal biochemical/enzymatic readouts of WDHD1 pathway engagement, such as DNA fiber assays for fork dynamics, nascent DNA synthesis (EdU), replisome integrity (chromatin-bound PCNA/MCM), and replication-stress checkpoints (pRPA/pCHK1), as well as binding/occupancy assays (e.g., ChIP-seq for WDHD1 or partner factors) where transcriptional regulation is implicated. These additions complement rather than contradict our reliance on shRNA. Together with the rescue data, they are expected to further strengthen the specificity and causal link between *WDHD1* depletion and the observed phenotypes. Furthermore, we acknowledge that single-pulse EdU assays primarily report DNA-synthesis output and cannot resolve whether WDHD1 knockdown impairs S-phase entry versus fork progression or completion. In line with Luo et al. on imaging-based replication progression and with Wu et al. on the need for direct interaction evidence to support mechanistic specificity [38,39], we will incorporate pulse–chase EdU/BrdU paradigms and dual-label DNA fiber assays (CldU/IdU) to quantify origin firing, fork speed, and termination/stall rates, together with time-resolved imaging of PCNA and RPA foci dynamics and checkpoint readouts (e.g., pRPA/pCHK1). Where transcriptional regulation or factor occupancy is implicated, we will assess direct chromatin engagement using ChIP-qPCR, CUT&RUN/CUT&Tag, and complementary proximity ligation/Co-IP assays. These orthogonal validations complement, rather than contradict, our EdU-based conclusion that *WDHD1* depletion reduces replication output; they are expected to refine the kinetics and mechanism by distinguishing effects on S-phase entry versus elongation and by mapping the relevant interaction landscape. Additionally, we recognize that annexin V–/PI primarily captures membrane externalization and benefits from orthogonal confirmation of executioner caspase activity. In line with Molnár et al. [40], we will complement annexin V–PI with immunoblotting for cleaved caspase 3 and cleaved PARP at matched time points. We note as a limitation that caspase 3/7 enzymatic assays were not included, and will incorporate time-resolved activity measurements and inhibitor rescue in future work. For resistance readouts, copy-number variation can confound interpretation, as highlighted by Ramalingam et al. [41]. Our perturbations were conducted at low MOI with puromycin selection and analyzed within early passages to minimize variable integration. Nonetheless, we will add qPCR/ddPCR-based vector/genomic copy-number assessment and normalization to future assays. Consistent with Li et al. on best practices for multi-omic housekeeping validation [42], we evaluated reference stability: qRT-PCR normalization used multiple candidate reference genes vetted by geNorm/NormFinder, immunoblotting employed total-protein normalization with verification that GAPDH/ACTB remained stable, and transcriptomic/proteomic data were scaled by median/global methods. These controls—and the planned orthogonal additions—enhance the robustness of our apoptosis and resistance conclusions and further de-risk technical confounders.

In conclusion, this study demonstrates that WDHD1 is upregulated in pancreatic cancer and regulates cell-cycle progression and apoptosis through the E2F1–WDHD1 axis by modulating the CDK4–cyclin D1 complex. While WDHD1 has been implicated in other malignancies such as breast cancer and lung cancer, its oncogenic role in pancreatic cancer has remained unexplored until now. Our findings not only expand the oncogenic spectrum of WDHD1 to pancreatic cancer but also reveal a novel transcriptional regulatory mechanism by E2F1, distinguishing it from previous reports in other cancer types. Furthermore, compared to established pancreatic cancer drivers such as KRAS and TP53, WDHD1 represents a previously unrecognized cell-cycle regulator that may serve as a potential therapeutic target. Given the limited efficacy of current KRAS-targeted therapies, targeting the E2F1–WDHD1 axis may offer a new avenue for combination strategies. These findings provide a rationale for developing WDHD1 inhibitors and highlight their potential clinical value in pancreatic cancer treatment.

## Figures and Tables

**Table 1:** shRNA sequence.

ShRNA	Sequence (5′ to 3′)
shWDHD1-F1	CCGGGCATCCTACTTGTGGTCGAATCTCGAGATTCGACCACAAGTAGGATGCTTTTTG
shWDHD1-R1	AATTCAAAAAGCATCCTACTTGTGGTCGAATCTCGAGATTCGACCACAAGTAGGATGC
shWDHD1-F2	CCGGCCCTGCTTCTTCGATTGTTTACTCGAGTAAACAATCGAAGAAGCAGGGTTTTTG
shWDHD1-R2	AATTCAAAAACCCTGCTTCTTCGATTGTTTACTCGAGTAAACAATCGAAGAAGCAGGG

## Data Availability

TCGA https://portal.gdc.cancer.gov/ and other data are contained within the article or Supplementary Materials.

## Supplementary Materials

The following supporting information can be downloaded at https://www.mdpi.com/article/10.3390/curroncol33040186/s1, Figure S1: *WDHDl* gene was identified through WGCNA analysis; Figure S2: Overexpression of WDHDl promotes the proliferation of pancreatic cancer cells; Figure S3: E2Fl is a possible transcription factor for WDHDl; Figure S4: The effects of E2Fl-WDHD1 on Cyclin D1 and CDK4 at the mRNA level; Figure S5: Effect of WDHDl knockdown on apoptosis-related molecules *in vivo*; Table S1: Primers used in the qPCR experiments; File S1: Unedited images for all blots and gels in the manuscript associated with figures and supplementary figures; Data S1: *WDHD1* promoter sequence and primer sequence for Chip-qPCR.

## Abbreviations

BZA, bazedoxifene; CH3, (E)-5-(3,4-dichlorostyryl)benzo[c][1,2]oxaborol-1(3H)-ol; EdU, 5-ethynyl-2′-deoxyuridine; HMG, high-mobility group; PBS, phosphate-buffered saline; PEI, polyethylenimine; PFA, paraformaldehyde; PI, propidium iodide; qPCR, quantitative PCR; qRT-PCR, quantitative real-time polymerase chain reaction; RT-PCR, reverse-transcription PCR; SD, standard deviation; STR, short tandem repeat; TCGA, The Cancer Genome Atlas; TPD, targeted protein degradation; WDHD1, WD repeat and HMG-box DNA-binding protein 1.
