# Supplementary material for: E2F1-Driven WDHD1 Transcription Enhances Cell-Cycle Progression and Promotes Pancreatic Cancer Progression
Source: Curr Oncol. 2026 Mar 26;33(4):186. doi: 10.3390/curroncol33040186 (PMC13115010; doi:10.3390/curroncol33040186)
Supplement: Supplementary file 1 [file curroncol-33-00186-s001.zip › Supplementary File S1.pdf]

E2F1-driven WDHD1 transcription enhances cell cycle progression and promotes pancreatic cancer progression

unedited images for all blots and gels in the manuscript associated with Figures and Supplementary figures

Full unedited gel for
Figure 1G

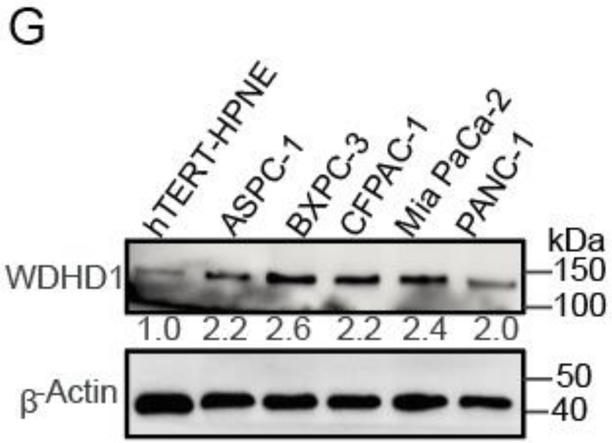

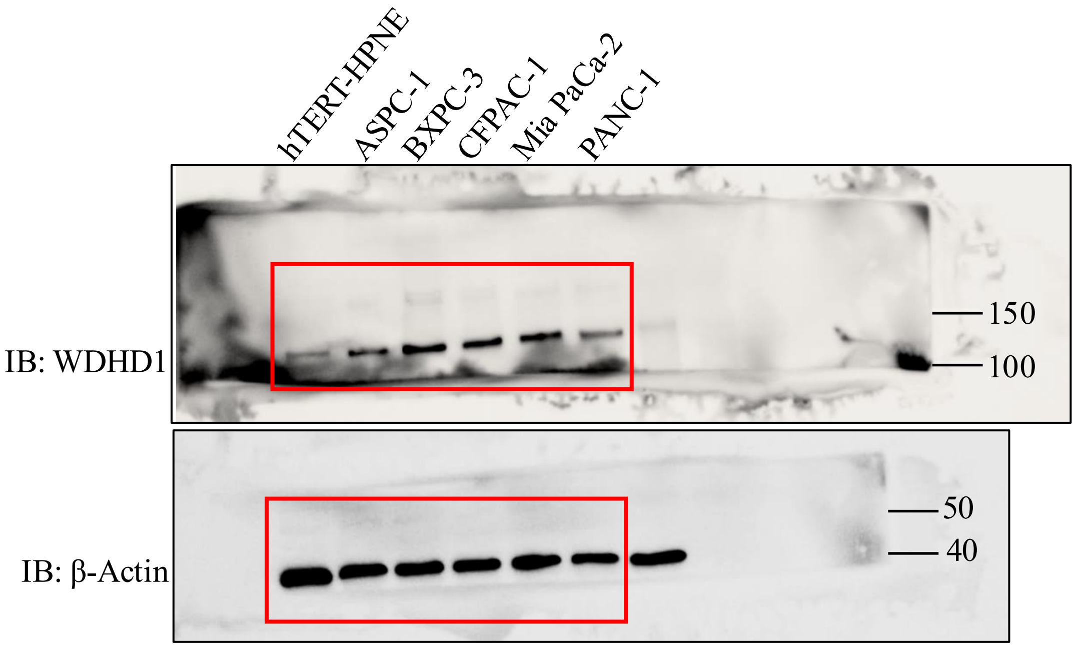

The blots come from the same batch of sample

Full unedited gel for
Figure 1H

H

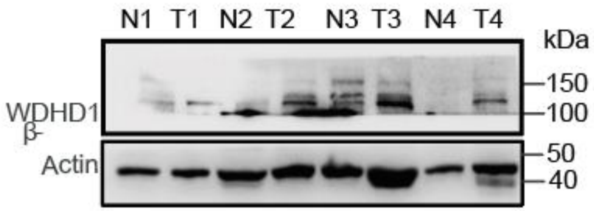

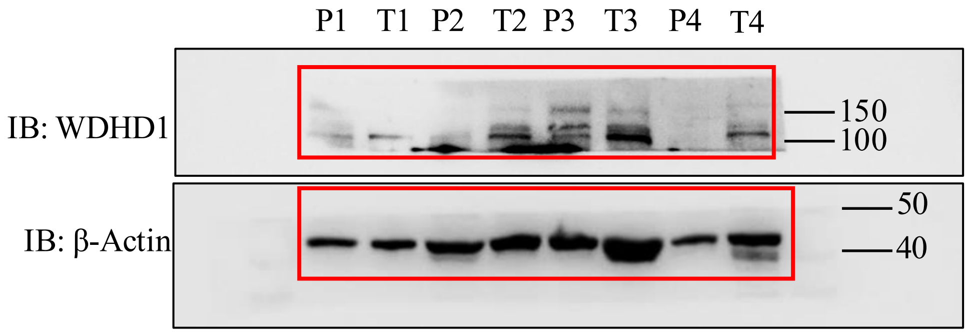

The blots come from the same batch of sample

Full unedited gel for Figure 2A

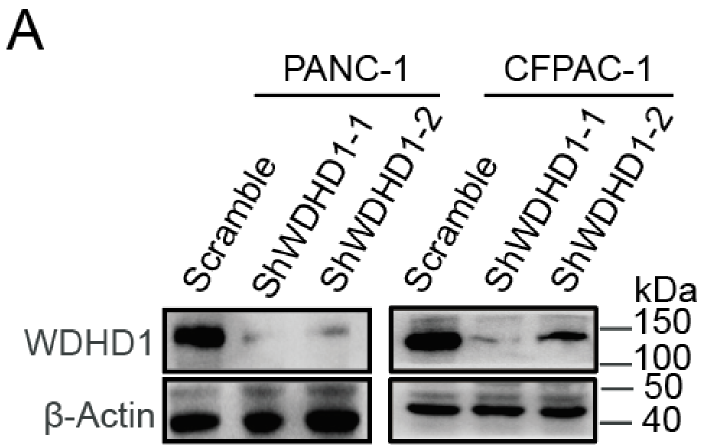

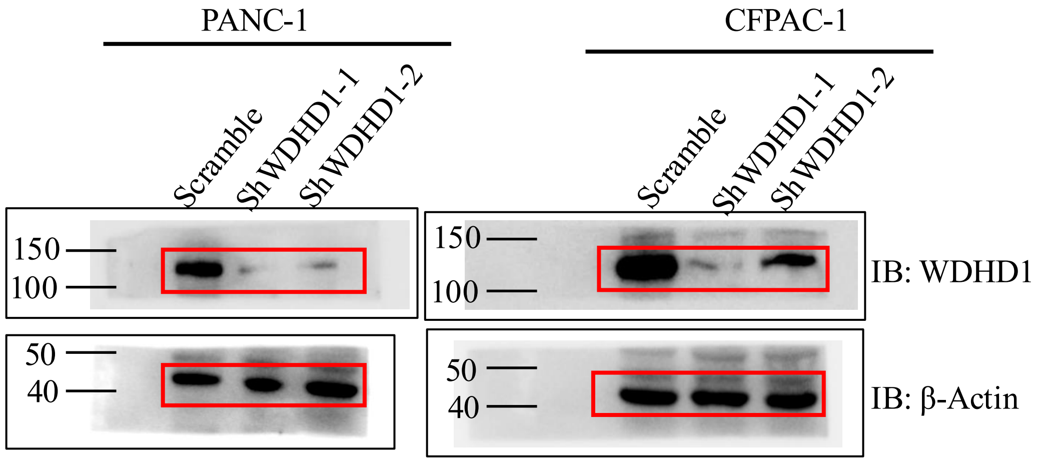

The blots come from the same batch of sample

Full unedited gel for
Figure 5A

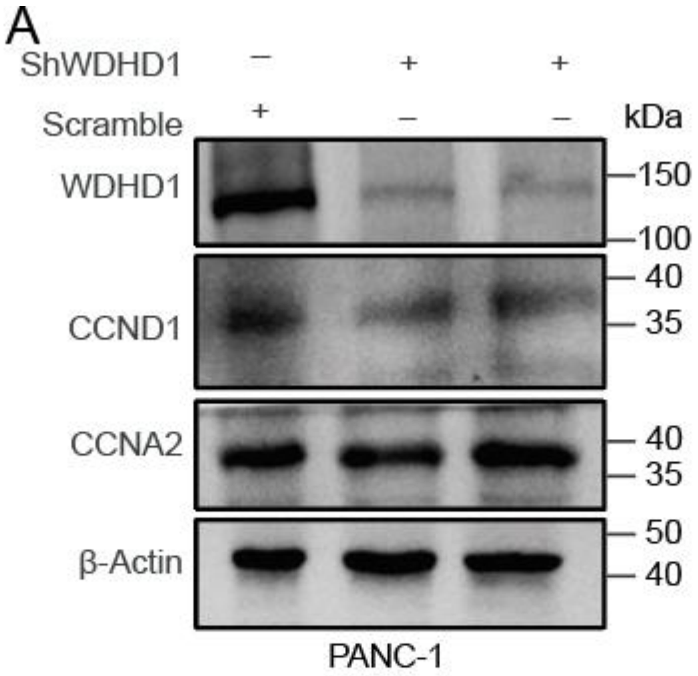

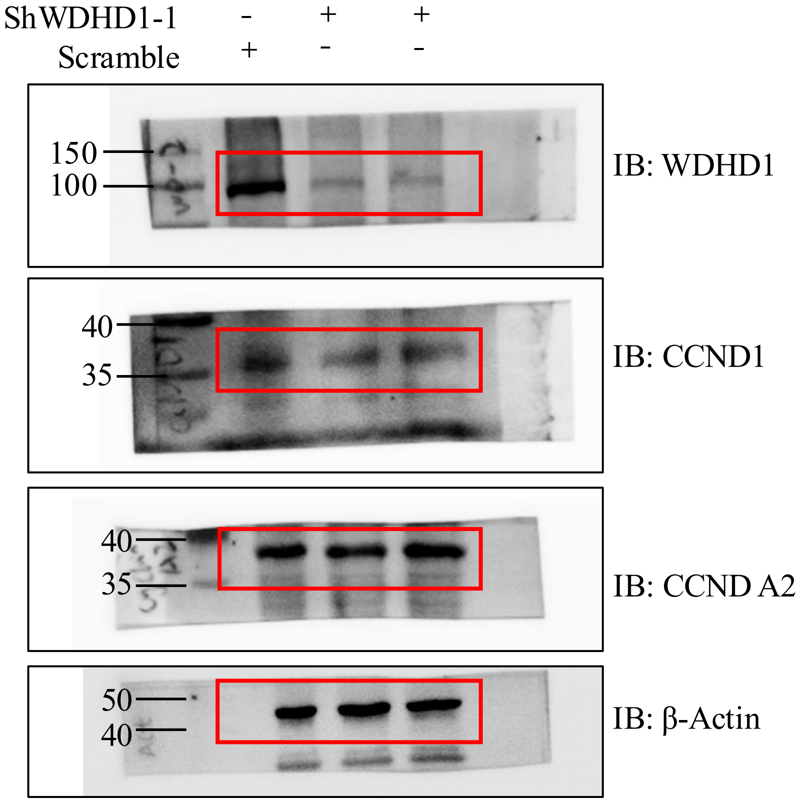

The blots come from the same batch of sample

Full unedited gel for
Figure 5B

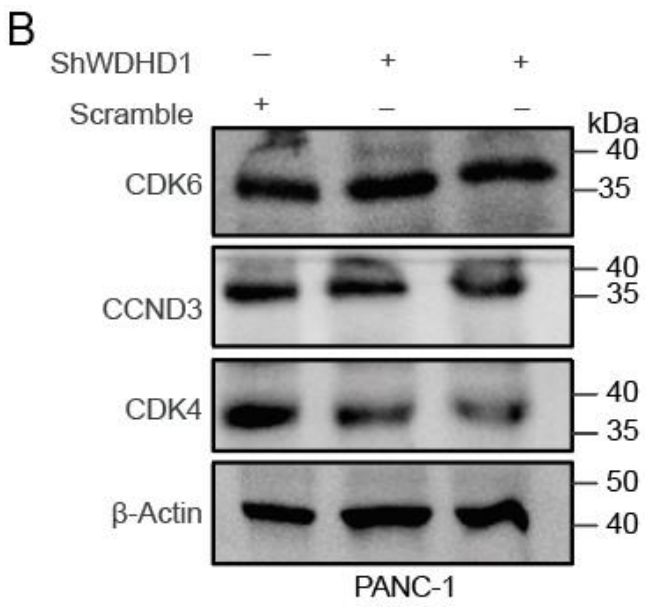

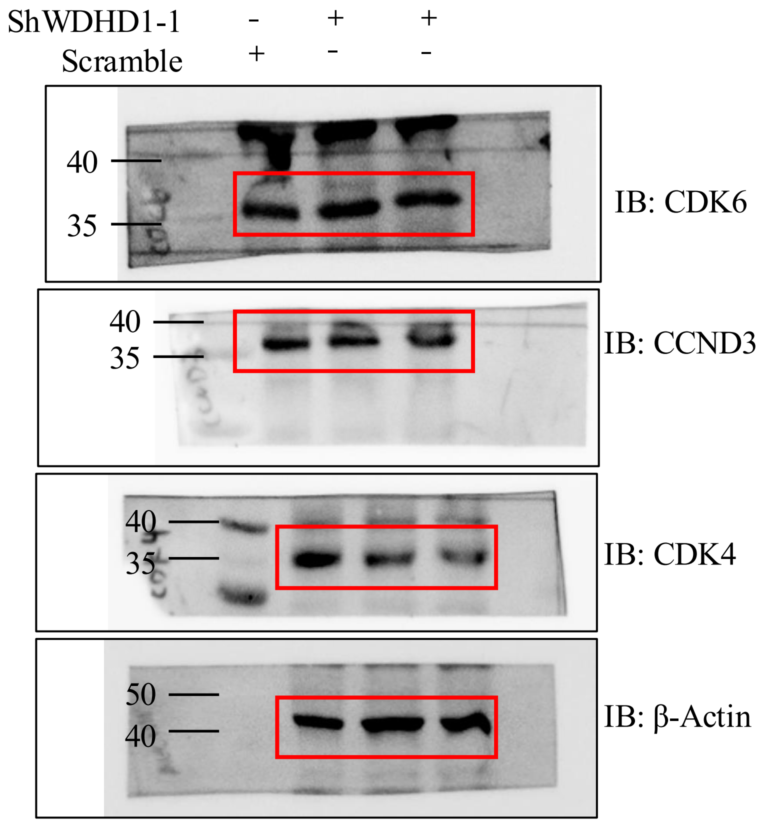

The blots come from the same batch of sample

Full unedited gel for Figure 5C

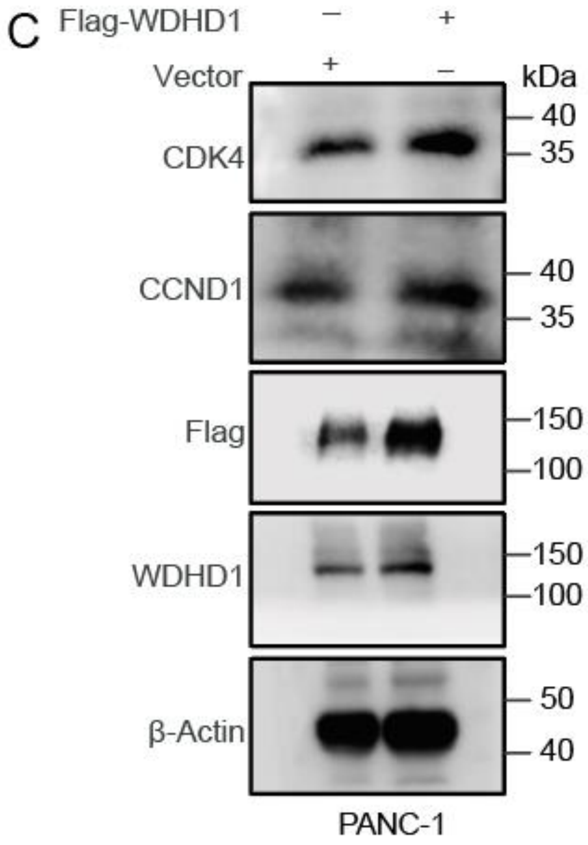

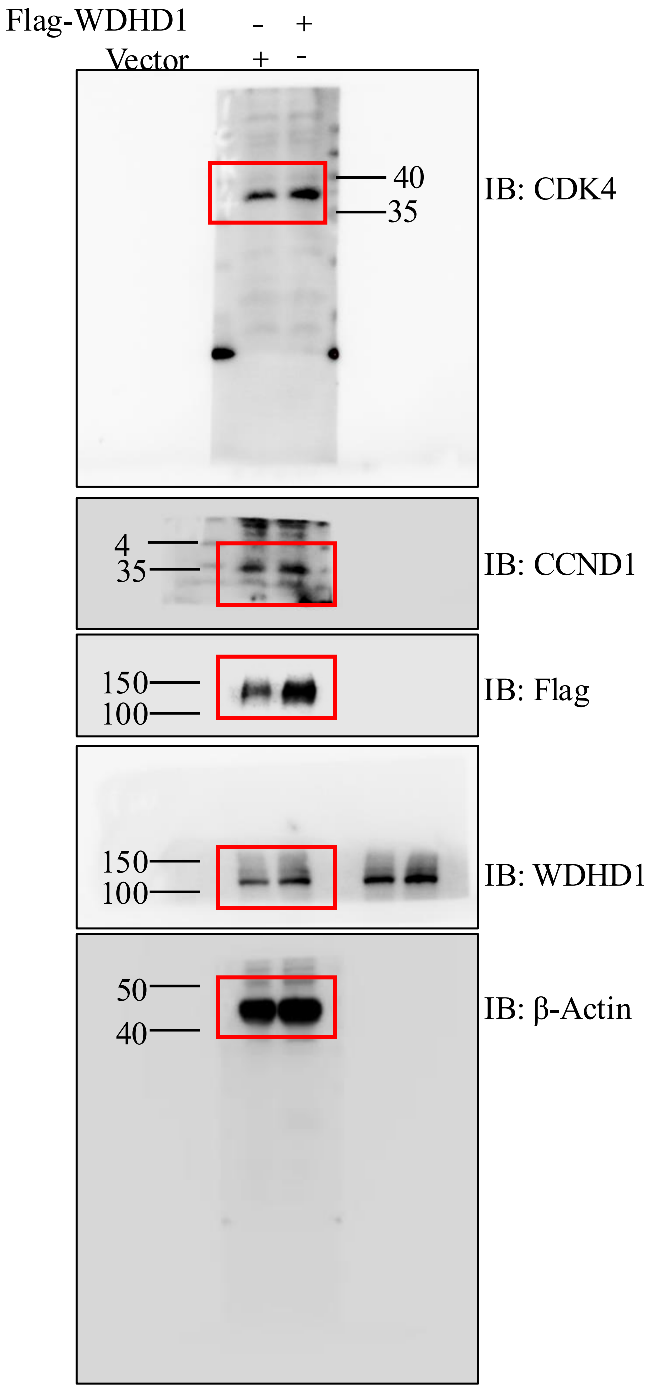

The blots come from the same batch of sample

Full unedited gel for
Figure 5F

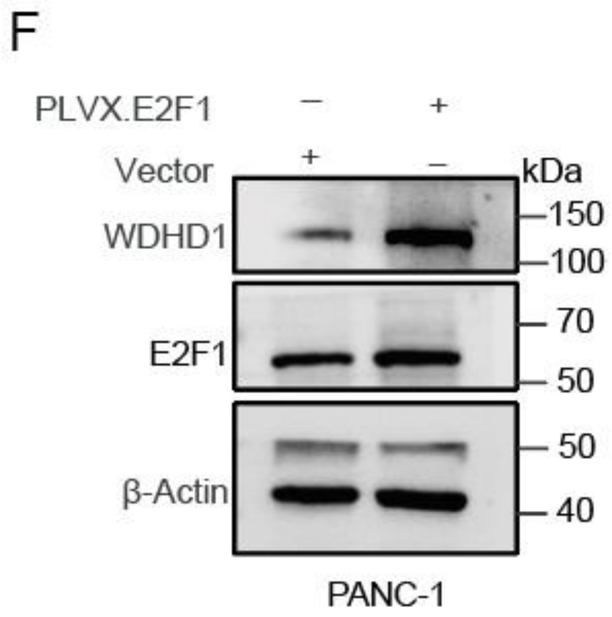

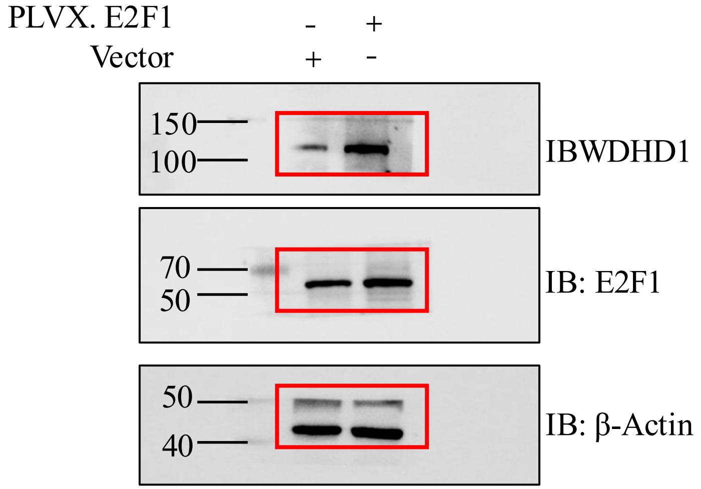

The blots come from the same batch of sample

Full unedited gel for
Figure 5G

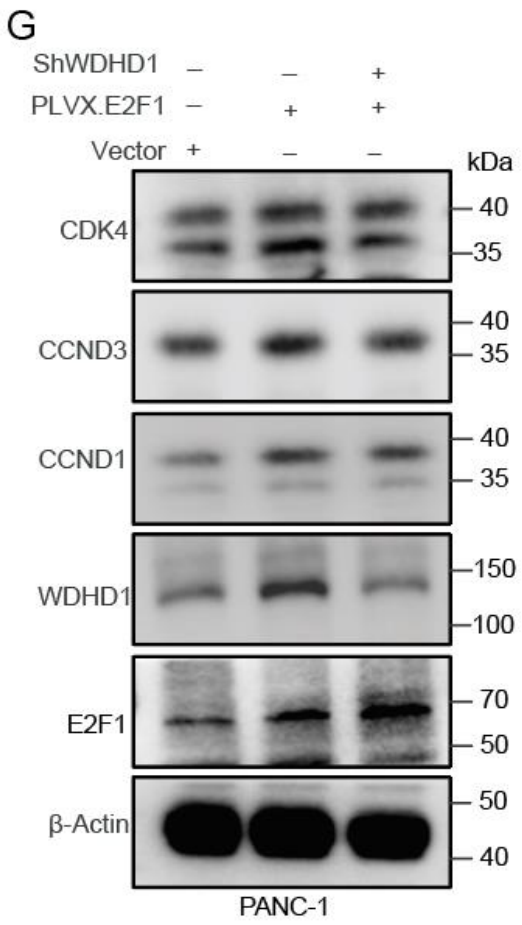

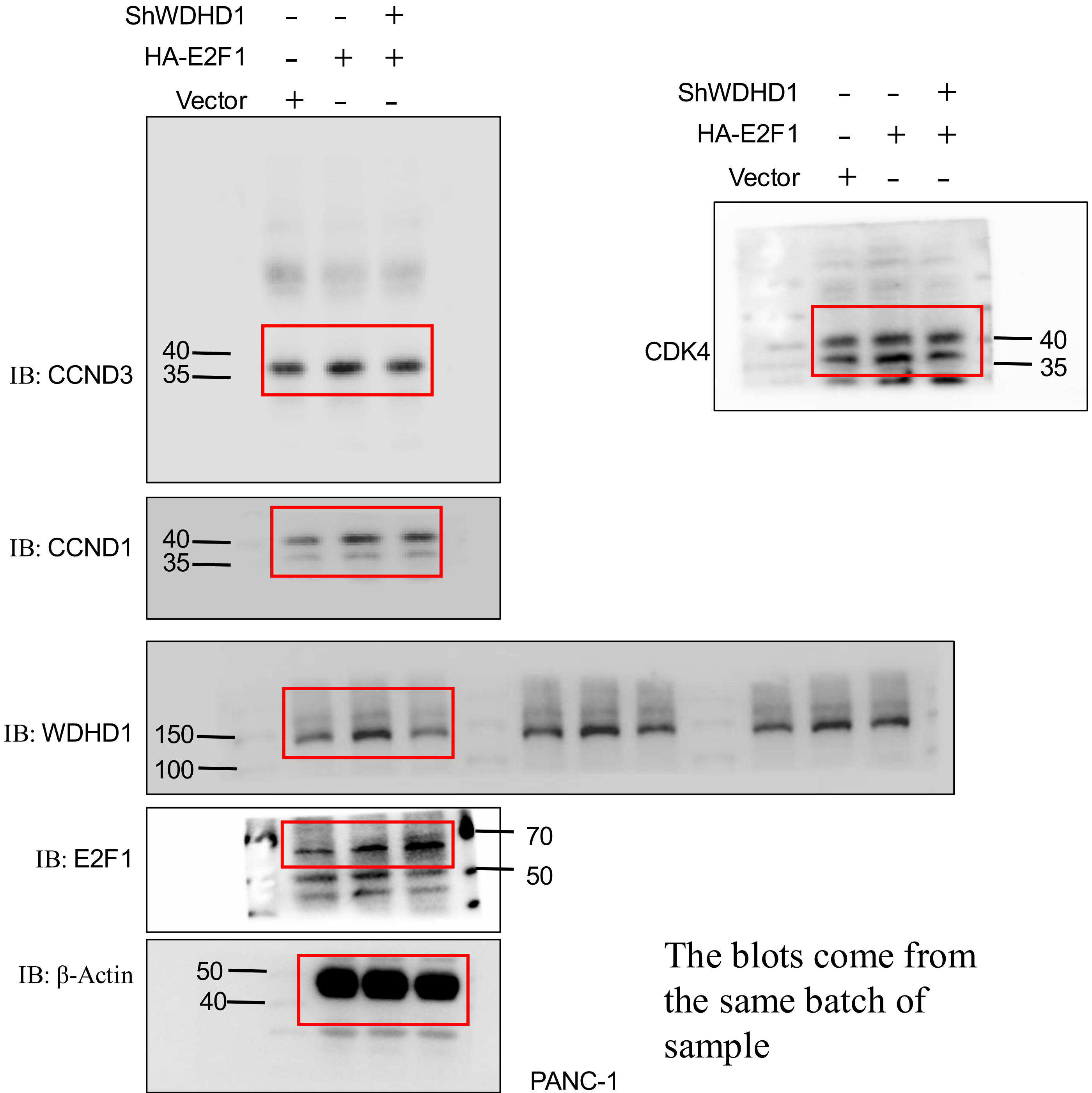

The blots come from the same batch of sample

Full unedited gel for
Figure 6A

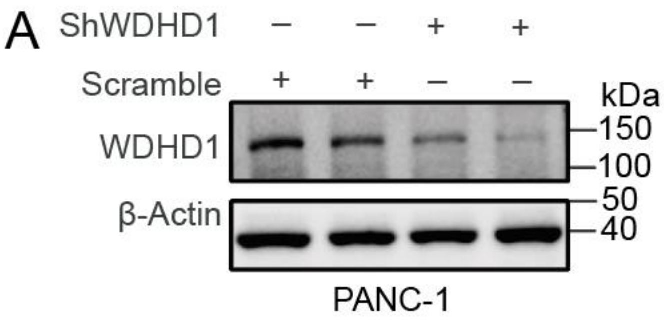

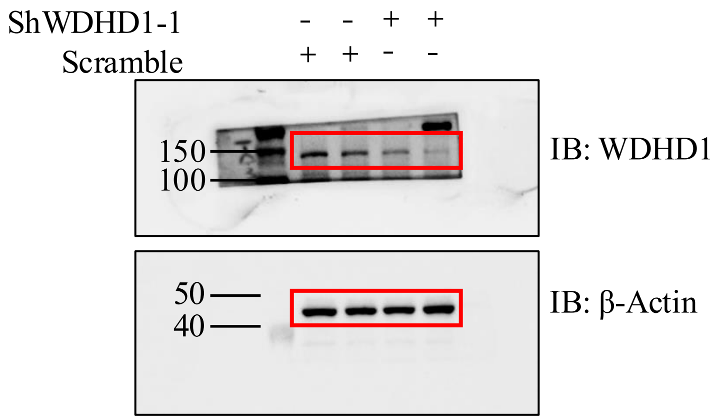

The blots come from the same batch of sample

Full unedited gel for
Figure 6F

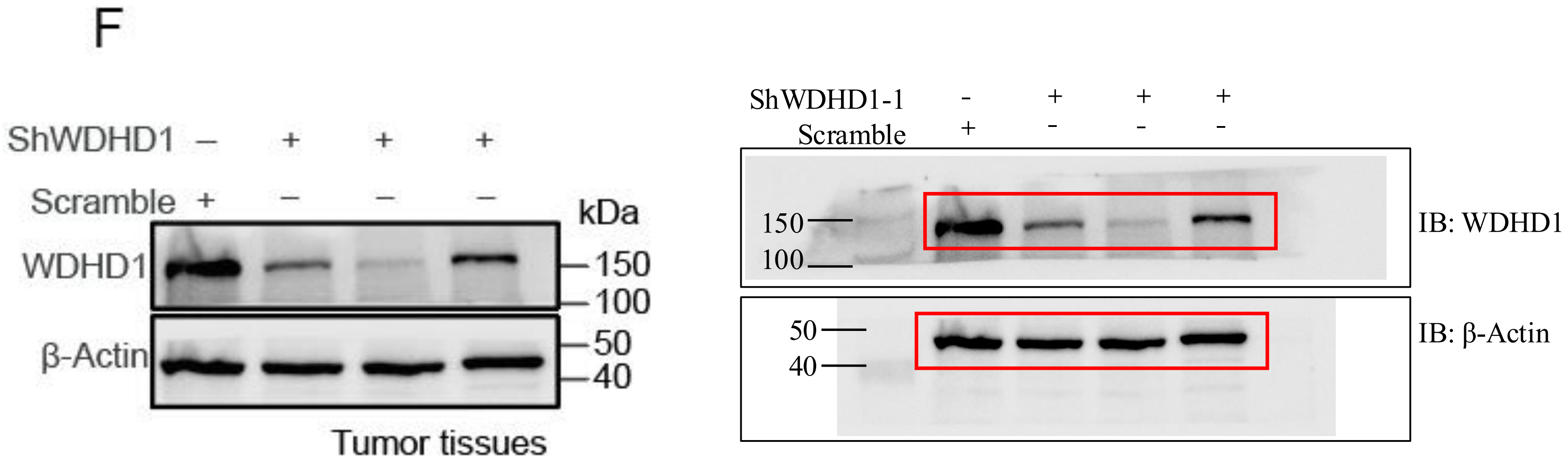

The blots come from the same batch of sample

Full unedited gel for
Suppplementary Figure 2A

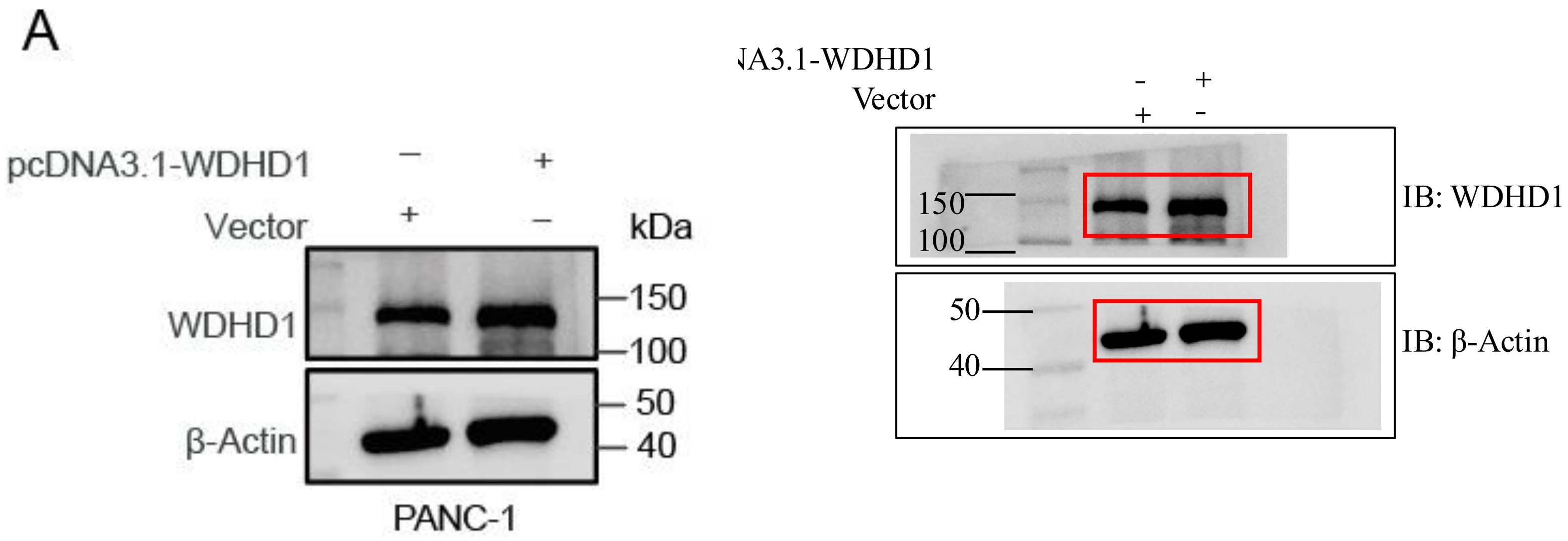

The blots come from the same batch of sample
