# Supplementary material for: E2F1-Driven WDHD1 Transcription Enhances Cell-Cycle Progression and Promotes Pancreatic Cancer Progression
Source: Curr Oncol. 2026 Mar 26;33(4):186. doi: 10.3390/curroncol33040186 (PMC13115010; doi:10.3390/curroncol33040186)
Supplement: Supplementary file 1 [file curroncol-33-00186-s001.zip › supplementay materials.pdf]

Article

E2F1-Driven WDHD1 Transcription Enhances Cell-Cycle Progression and Promotes Pancreatic Cancer Progression

Xiaojuan Yang [1,2,†], Zhiwei Zhang [1,3,†], Shuangjuan Lv [2], Buzhe Zhang [2], Xue Tao [2], Chang Liu [1,4,*] and Qing Zhu [1,*]

[1] Department of Abdominal Oncology, West China Hospital, Sichuan University, Chengdu 610041, China; y3532388972@163.com (X.Y.); 12418716@zju.edu.cn (Z.Z.)

[2] Laboratory of Hepatic AI Translation, Frontiers Science Center for Disease-Related Molecular Network, West China Hospital, Sichuan University, Chengdu 610041, China; lvshuangjuan@wchscu.cn (S.L.); 2024224020203@stu.scu.edu.cn (B.Z.); 2024224200031@stu.scu.edu.cn (X.T.)

[3] Key Laboratory of Cancer Prevention and Intervention, Department of Colorectal Surgery and Oncology, Ministry of Education, the Second Affiliated Hospital, Zhejiang University School of Medicine, Hangzhou 322000, China

[4] Intervention Center, West China Hospital, Sichuan University, No. 37 Guoxue Alley, Chengdu 610041, China

* Correspondence: drliuchang@wchscu.cn (C.L.); yy1432268212@163.com (Q.Z.)

† These authors contributed equally to this work.

Table S1. Primers used in the qPCR experiments.

Gene Symbol	GenBank Accession numbers	Sequence (5' to 3')	Amplicon size (bp)	Annealing temperatures (°C)
WDHD1	NM_007086.4	Forward primer: CATGGCATCCTACTTGTGGTC<br>Reverse primer: AAGGGATCTCAACTGCATTGTC	204	60
CyclinD1	NM_053056.3	Forward primer: TGTCCTACTACCGCCTCACA<br>Reverse primer: CAGGGCTTCGATCTGCTC	92	60
CDK4	NM_000075.4	Forward primer: TCAGCACAGTTCGTGAGGTG<br>Reverse primer: GTCCATCAGCCGGACAACAT	77	60
E2F1	NM_005225.3	Forward primer: CATCCCAGGAGGTCACTTCTG<br>Reverse primer: GACAACAGCGGTTCTTGCTC	145	60
GAPDH	NM_001289746.2	Forward primer: GTCTCCTCTGACTTCAACAGCG<br>Reverse primer: ACCACCCTGTTGCTGTAGCCAA	131	60

We have verified the specificity of all qPCR primers (including WDHD1 and reference gene primers) using NCBI's Primer-BLAST tool (https://www.ncbi.nlm.nih.gov/tools/primer-blast/). The screen-shot results are as follows:

WDHD1:

	Sequence (5'->3')	Length	Tm	GC%	Self complementarity	Self 3' complementarity
**Forward primer**	CATGGCATCCTACTTGTGGTC	21	58.71	52.38	5.00	1.00
**Reverse primer**	AAGGGATCTCAACTGCATTGTC	22	58.65	45.45	6.00	2.00

Products on target templates

>NM_007086.4 Homo sapiens WD repeat and HMG-box DNA binding protein 1 (WDHD1), transcript variant 1, mRNA

```
product length = 204
Forward primer  1    CATGGCATCCTACTTGTGGTC  21
Template        927  .....................  947

Reverse primer  1     AAGGGATCTCAACTGCATTGTC  22
Template        1130  ......................  1109
```

CyclinD1 :

	Sequence (5'->3')	Length	Tm	GC%	Self complementarity	Self 3' complementarity
**Forward primer**	TGTCCTACTACCGCCTCACA	20	59.96	55.00	3.00	0.00
**Reverse primer**	CAGGGCTTCGATCTGCTC	18	57.57	61.11	4.00	3.00

Products on target templates

>NM_053056.3 Homo sapiens cyclin D1 (CCND1), mRNA

```
product length = 92
Forward primer  1    TGTCCTACTACCGCCTCACA  20
Template        829  ....................  848

Reverse primer  1    CAGGGCTTCGATCTGCTC  18
Template        920  ..................  903
```

CDK4:

	Sequence (5'->3')	Length	Tm	GC%	Self complementarity	Self 3' complementarity
**Forward primer**	TCAGCACAGTTCGTGAGGTG	20	60.25	55.00	5.00	2.00
**Reverse primer**	GTCCATCAGCCGGACAACAT	20	60.39	55.00	5.00	2.00

Products on target templates

>NM_000075.4 Homo sapiens cyclin dependent kinase 4 (CDK4), mRNA

```
product length = 77
Forward primer  1    TCAGCACAGTTCGTGAGGTG  20
Template        307  ....................  326

Reverse primer  1    GTCCATCAGCCGGACAACAT  20
Template        383  ....................  364
```

E2F1:

	Sequence (5'->3')	Length	Tm	GC%	Self complementarity	Self 3' complementarity
**Forward primer**	CATCCCAGGAGGTCACTTCTG	21	59.79	57.14	5.00	5.00
**Reverse primer**	GACAACAGCGGTTCTTGCTC	20	59.76	55.00	6.00	5.00

Products on target templates

>NM_005225.3 Homo sapiens E2F transcription factor 1 (E2F1), mRNA

```
product length = 145
Forward primer  1     CATCCCAGGAGGTCACTTCTG  21
Template        1056  .....................  1076

Reverse primer  1     GACAACAGCGGTTCTTGCTC  20
Template        1200  ....................  1181
```

GAPDH:

	Sequence (5'->3')	Length	Tm	GC%	Self complementarity	Self 3' complementarity
**Forward primer**	GTCTCCTCTGACTTCAACAGCG	22	60.92	54.55	4.00	2.00
**Reverse primer**	ACCACCCTGTTGCTGTAGCCAA	22	64.41	54.55	4.00	4.00

Products on target templates

>NM_001289746.2 Homo sapiens glyceraldehyde-3-phosphate dehydrogenase (GAPDH), transcript variant 4, mRNA

```
product length = 131
Forward primer  1     GTCTCCTCTGACTTCAACAGCG  22
Template        1160  ......................  1181

Reverse primer  1     ACCACCCTGTTGCTGTAGCCAA  22
Template        1290  ......................  1269
```

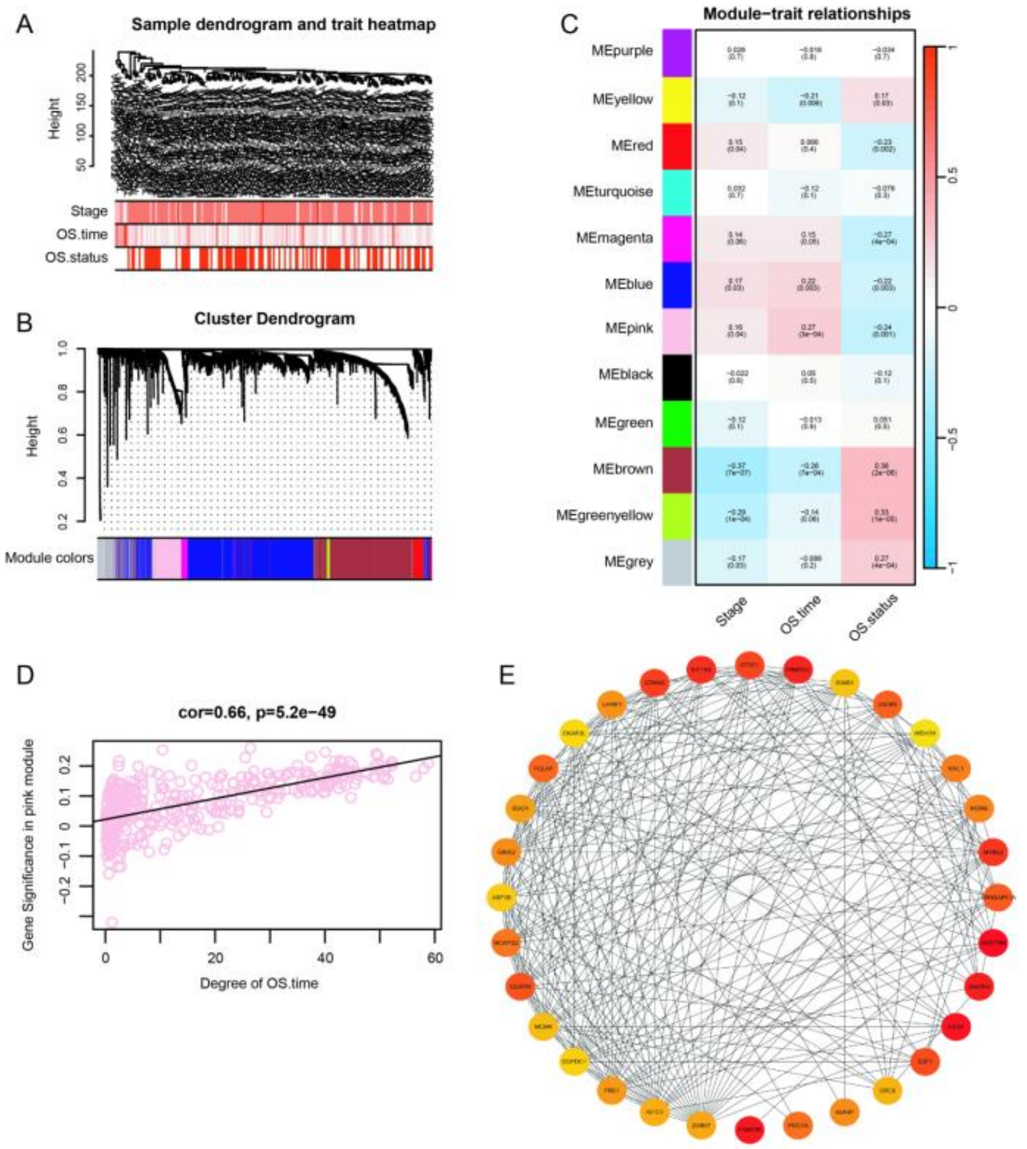

Figure S1. WDHD1 gene was identified through WGCNA analysis.
(A) Tree diagram of 178 pancreatic cancer samples and their stages, survival time, and survival status in the TCGA database. (B) Division of 20,000 genes into 12 gene modules based on soft thresholds. (C) Correlation analysis results between 12 gene modules and 3 phenotypes. (D) Correlation analysis results between the pink gene module and patient survival. (E) Display of the correlation network between 32 node genes.

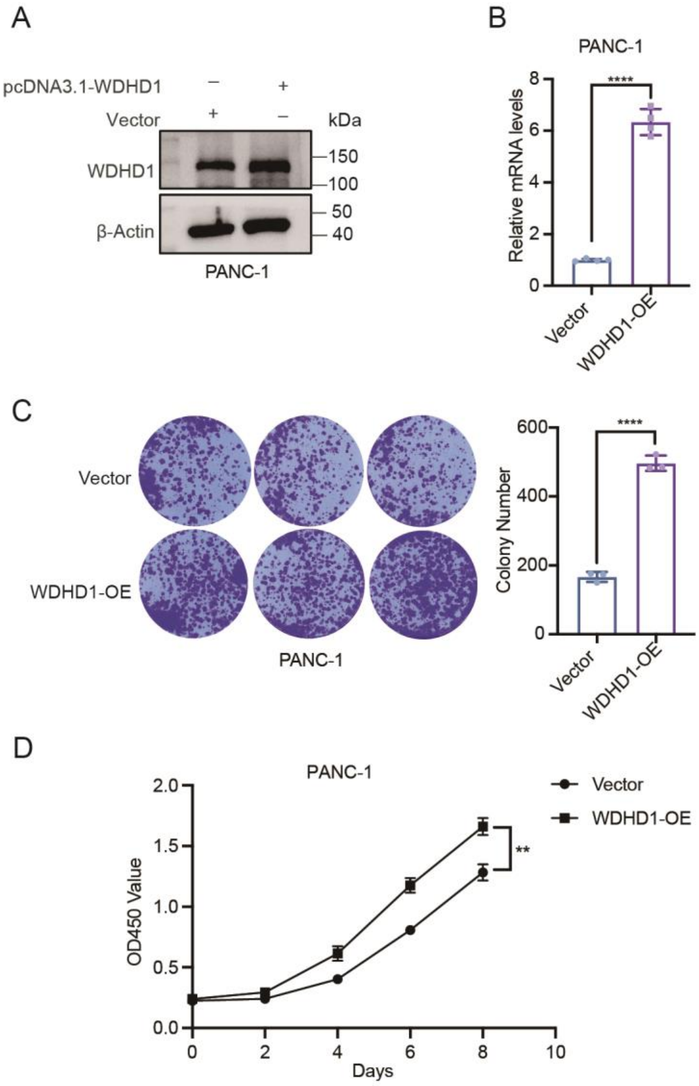

Figure S2. Overexpression of WDHD1 promotes the proliferation of pancreatic cancer cells.

(A) Western blot results confirming overe xpression of WDHD1 in the PANC-1 cell line. Western blot verification of the protein knockdown level of WDHD1 in the pancreatic cancer cell line. Western blot verification of the protein overexpression level of WDHD1 in the pancreatic cancer cell line. β-actin serves as a loading control; Representative images were selected from three independent experiments. (B) qPCR results showing overexpression of WDHD1 in the PANC-1 cell line. qPCR verification of the RNA overexpression level of WDHD1 in pancreatic cancer cell lines. n = 3 (independent biological replicates). Statistical significance: *$p < 0.05$, **$p < 0.01$, ***$p < 0.001$, ****$p < 0.0001$ (Student's *t*-test). (C) Overexpression of WDHD1 in the PANC-1 cell line increased the number of cell clones. Representative images were selected from three independent experiments; Statistical significance: *$p < 0.05$, **$p < 0.01$, ***$p < 0.001$, ****$p < 0.0001$ (Student's *t*-test). (D) Overexpression of WDHD1 promotes cell proliferation in the PANC-1 cell line. n = 3 (independent biological replicates). Statistical significance: *$p < 0.05$, **$p < 0.01$, ***$p < 0.001$, ****$p < 0.0001$ (Student's *t*-test).

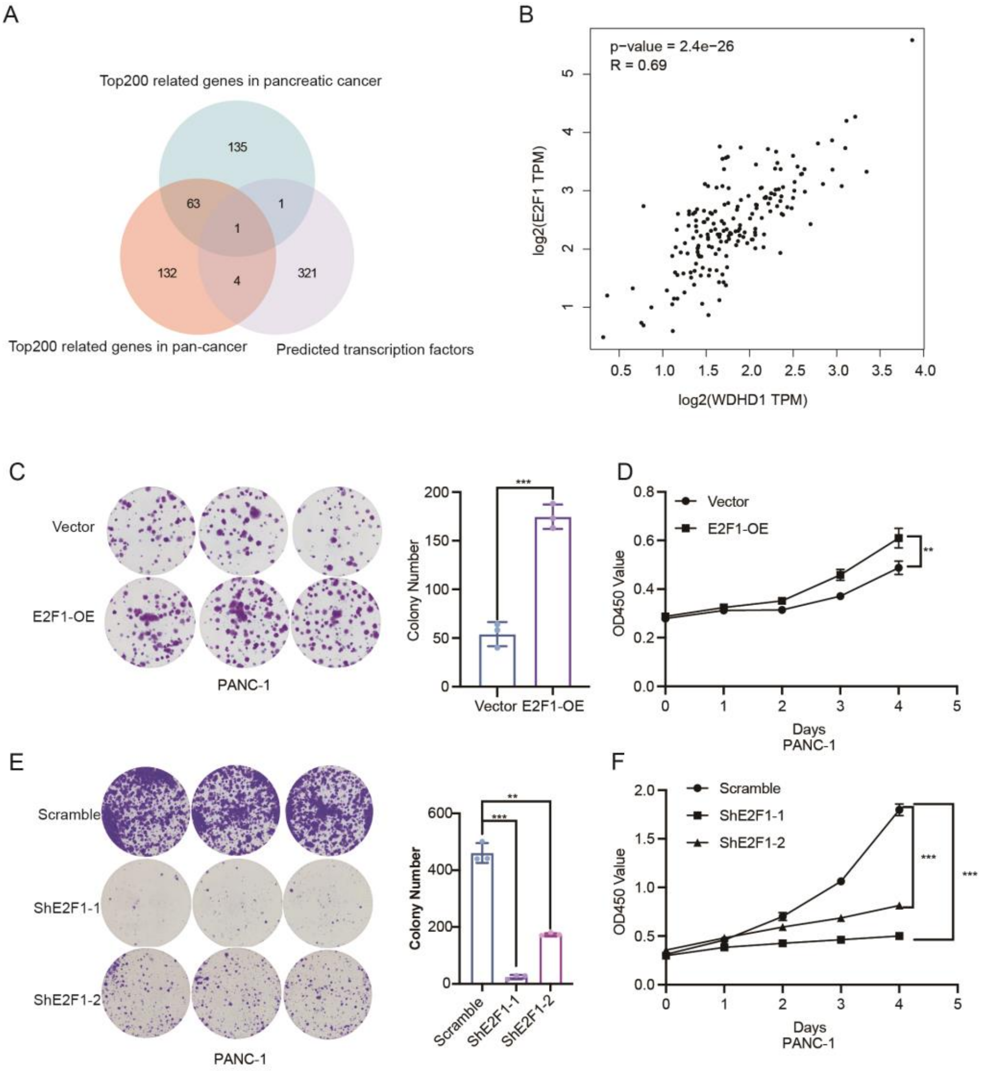

Figure S3. E2F1 is a possible transcription factor for WDHD1.
(A) Venn diagram of three gene sets. (B) Positive correlation between WDHD1 and E2F1 expression in pancreatic cancer. (C-D) Results of the plate cloning experiment and CCK8 experiment for overexpression of E2F1. Representative images were selected from three independent experiments; Statistical significance: *p < 0.05, **p < 0.01, ***p < 0.001, ****p < 0.0001 (Student's *t*-test). (E-F) Results of the plate cloning experiment and CCK8 experiment for knockdown of E2F1. Representative images were selected from three independent experiments; Statistical significance: *p < 0.05, **p < 0.01, ***p < 0.001, ****p < 0.0001 (Student's *t*-test).

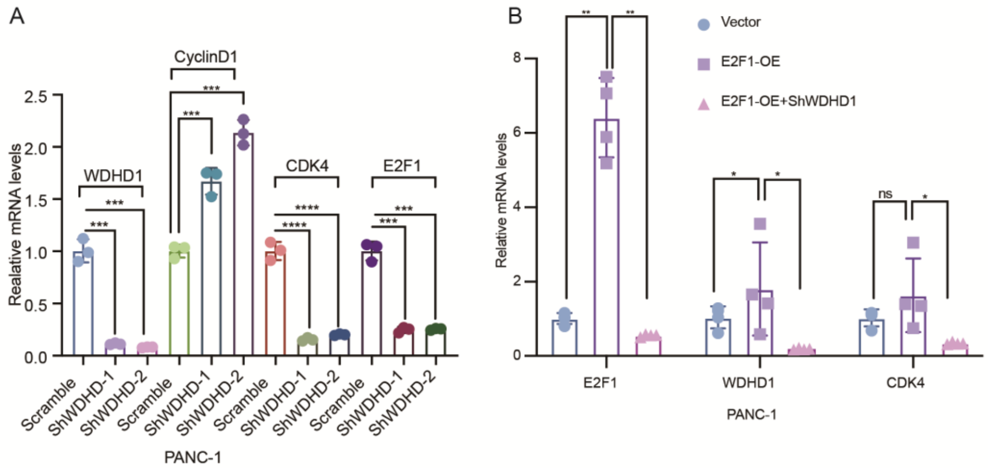

Figure S4. The effects of E2F1-WDHD1 on Cyclin D1 and CDK4 at the mRNA level.
(A) The effect of knocking down WDHD1 on CDK4 and E2F1 at the RNA level. n = 3 (independent biological replicates); Summary statistics for each experimental group were reported as mean ± SD; Statistical significance: *$p < 0.05$, **$p < 0.01$, ***$p < 0.001$, ****$p < 0.0001$ (Student's t-test). (B) The impact of knocking down WDHD1 after overexpression of E2F1 on CDK4 at the RNA levels. n = 4 (independent biological replicates); Summary statistics for each experimental group were reported as mean ± SD; Statistical significance: *$p < 0.05$, **$p < 0.01$, ***$p < 0.001$, ****$p < 0.0001$ (Student's t-test).

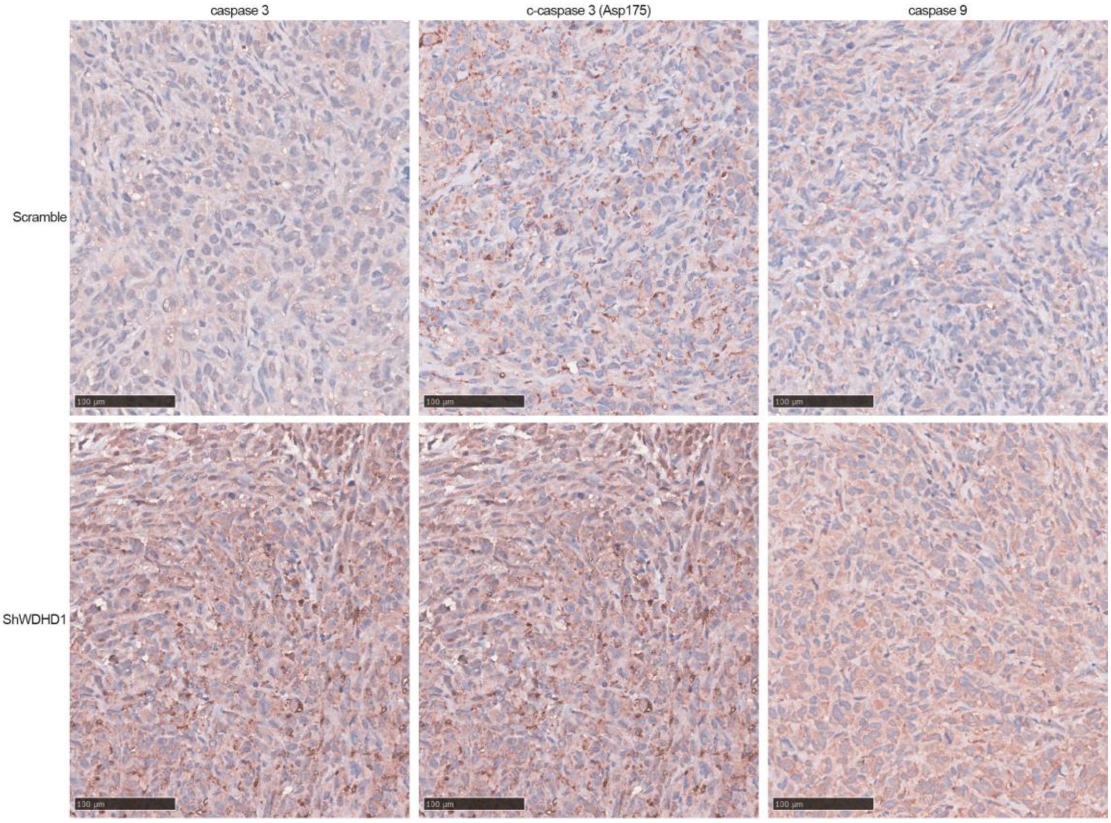

Figure S5. Effect of WDHD1 knockdown on apoptosis-related molecules *in vivo*. Representative immunohistochemistry images and quantification of caspase-3, cleaved caspase-3 (c-caspase 3, Asp175), and caspase-9 in subcutaneous tumor tissues from mice. Scale bar, 100 μm.
