# Supplementary figures and images for: E2F1-Driven WDHD1 Transcription Enhances Cell-Cycle Progression and Promotes Pancreatic Cancer Progression
Source: Curr Oncol. 2026 Mar 26;33(4):186. doi: 10.3390/curroncol33040186 (PMC13115010; doi:10.3390/curroncol33040186)

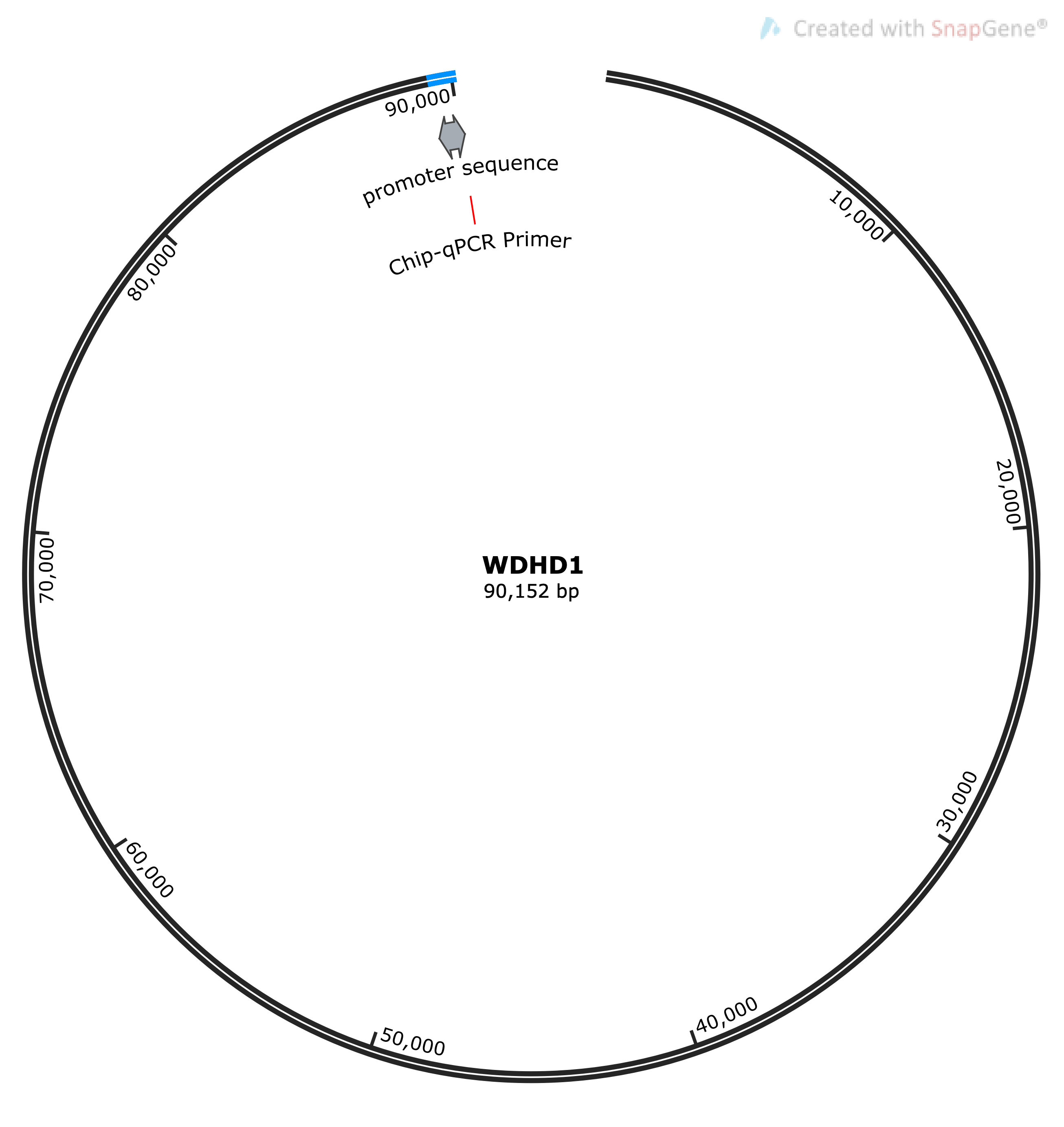

Supplement: Supplementary file 1 [file curroncol-33-00186-s001.zip › supplementary Data S1.tiff]
